# Prevalence of the Burden of Diseases Causing Visual Impairment and Blindness in South Africa in the Period 2010–2020: A Systematic Scoping Review and Meta-Analysis

**DOI:** 10.3390/tropicalmed7020034

**Published:** 2022-02-21

**Authors:** Zamadonda Nokuthula Xulu-Kasaba, Chester Kalinda

**Affiliations:** 1 Discipline of Optometry, School of Health Sciences, University of KwaZulu-Natal, University Road, Durban 4001, South Africa; 2 Bill and Joyce Cummings Institute of Global Health, University of Global Health Equity (UGHE), Kigali P.O. Box 6955, Rwanda; kalindac@ukzn.ac.za; 3 Institute of Global Health Equity Research (IGHER), University of Global Health Equity (UGHE), Kigali P.O. Box 6955, Rwanda; 4 Department of Public Health, School of Nursing and Public Health, Howard College Campus, University of KwaZulu-Natal, Durban 4001, South Africa

**Keywords:** visual impairment, cataract, diabetic retinopathy, glaucoma, refractive error, ocular disease, South Africa, eye health, eye care

## Abstract

The prevalence of visual impairment (VI) continues to rise, despite efforts to reduce it. The burden of disease negatively impacts the quality of life, education opportunities, and other developments in various communities. Henceforth, this study aimed to determine and quantify the major causes of VI in South Africa, to ensure accurate interventions in addressing them and to reduce the burden of ocular disease in that context. A systematic scoping review was conducted to map evidence on VI and ocular diseases, using the PRISMA-P guidelines. English studies were searched for on PubMed, Google Scholar, and EBSCOhost using various search terms. The eligible articles underwent screening and ultimately data extraction to identify major causes of VI in South Africa. A meta-analysis further resulted in pooled prevalence estimates (PPE) using the Inverse Variance Heterogeneity (IVhet) model. Of the 13,527 studies screened at three levels, 10 studies met the inclusion criteria for the final review; however, 9 studies were eligible for quality assessment performed by two independent reviewers. The quality index for the included studies was 71.1%. The prevalence of VI was 2% for blindness and 12% for moderate and severe visual impairment (MSVI). Pooled prevalence identified uncorrected refractive error (URE) (43%), cataract (28%), glaucoma (7%), and diabetic retinopathy (4%) as major causes of MSVI. The leading causes of blindness were untreated cataracts (54%), glaucoma (17%), and diabetic retinopathy (57%). Ocular diseases causing VI are avoidable and similar to those of low-to-middle income countries. MSVI were caused by URE, cataract, glaucoma, and diabetic retinopathy. Blindness was mainly caused by cataracts, glaucoma, and diabetic retinopathy. A strategic plan to manage these conditions would largely reduce the burden of VI in the country. Early screenings and interventions to maximize care at primary health levels would decrease the burden of avoidable blindness in the country significantly.

## 1. Introduction

With a global population of 217 million people having moderate to severe visual impairment (VI) and 36 million blind people [1], the challenge of reduced vision remains a serious public health challenge. Together with the International Agency for the Prevention of Blindness (IAPB), the “VISION 2020: Right to Sight” campaign was aimed at eliminating avoidable blindness and VI by the year 2020 [2]. However, the majority of the affected individuals are from low to middle-income countries [3], which are also heavily affected by other diseases such as neglected tropical diseases (NTDs), malaria, and tuberculosis, among others [4]. These health challenges in addition to visual impairment (VI) have far-reaching consequences that ultimately lead to reduced educational opportunities, minimal economic participation, increased rates of poverty, and reduced economic development amongst the disabled [1,5,6].

The World Health Organization (WHO) classifies reduced VI as moderate when presenting visual acuity (VA) ranges from 6/18 to 6/60, as severe with VA ranges from 6/60 to 3/60, and as blindness when the VA is worse than 3/60 [7]. Globally, disabilities like impaired vision negatively impact an individual’s health outcomes. As an enabler of development and growth [2,5], good vision improves the chances of employability, much needed in a region burdened by poverty such as Sub-Saharan Africa (SSA) [8,9]. 

South Africa is one of the most viable economies on the African continent, contributing about 25% of the continent’s Gross Domestic Product (GDP) [10,11]. Its population growth is 0.02% less than the average rate in the continent, a factor that further assists in containing poverty [8]. Of note, however, is that South Africa is the world’s most unequal country, owing to its apartheid legacy, where the gap between rich and poor continues to rise [12]. VI and blindness increase the possibility of poverty due to economical exclusion and reduced educational opportunities [3]. Evidence has confirmed that VI is the leading disability in South Africa [13], and its increase may have serious ramifications on both health and economic systems, as well as defer the attainment of the Sustainable Development Goal (SDG) 3.6 (adequate eye health) [14]. 

It is therefore important to understand the epidemiology of VI and blindness to improve eye health and prevent further increases in preventable blindness. Furthermore, the different types of ocular anomalies need to be known to ensure that correct strategies are put in place for adequate management. This systematic scoping review aimed to establish the prevalence of VI and blindness in SA. The study further sought to identify and quantify the major causes of vision impairment and blindness within the country. The resulting information would be useful for policymakers to improve decision making on programs for the prevention of blindness and provide guidance on human resource needs within eye health facilities in South Africa.

## 2. Methodology

Through a synthesis of empirical evidence, with a repeatable and consistent method [15], systematic scoping reviews contextualize knowledge and identify known and unknown information to answer a specific research question [16]. To adhere to a systematic process, improve rigor, and eliminate bias, the PRISMA reporting method was adopted in this study [17]. Using the Joanna Briggs methodology [15], this review identified the prevalence and causes of major vision impairment and blindness in South Africa, using peer-reviewed and grey literature. Article search was conducted on various databases following the five-step Arksey and O’Malley framework [18] and using recommendations by Levac et al. [19] to clarify how each step was followed. A meta-analysis of all included studies was conducted to present the results comprehensively. 

### 2.1. Search Strategy and Identification of Relevant Studies

The research question guiding the study was: What are the major causes of VI and blindness in South Africa? The study search was guided by the Population Concept Context (PCC) [15] model (Table 1). Searching for studies that met the stipulated inclusion and exclusion criteria was carried out as discussed below. 

***Inclusion criteria***

- Studies on human subjects across all ages
- Studies on eye diseases, ocular conditions, refractive error, and visual impairment
- Primary studies, published in English
- Studies published in the period 2010 to 2020
- Studies conducted within South Africa

***Exclusion criteria***

- Reviews and other studies that were not primary
- Studies on animals
- Incomplete studies
- Studies that did not quantify VI and Blindness prevalence

Databases searched for suitable articles included PubMed, Google Scholar, and EBSCOhost databases: Health Source: Nursing/Academic Edition, Health source–Consumer, CINAHL, and Academic Search Complete. Primary studies that quantified disease prevalence were included in this systematic review. Following this search, unpublished rapid studies were further searched for in relevant repositories. These were included as they gave quantitative information on the burden of VI and blindness.

To ensure the feasibility of the study and the appropriateness of the keywords, the search strategy was piloted, and feasibility was confirmed prior to the commencement of the study.

***Search terms and study selection***

“Prevalence of visual impairment OR Prevalence of Blindness OR Prevalence of Avoidable blindness OR Epidemiology of Visual Impairment OR Vision Impairment AND South Africa AND 2010–2020”.

Medical Subject Heading (MeSH) terms, which operate like synonyms, were used to further improve the search of studies. MeSH terms for the leading global causes of VI and blindness, given in Table 1, were further used to improve the study search [16]. To improve specificity, Boolean operators “AND” (to specify the time frame and context of the study) and “OR” (to separate the different visual anomalies and ocular diseases) were used in this study. 

**Figure 1:**
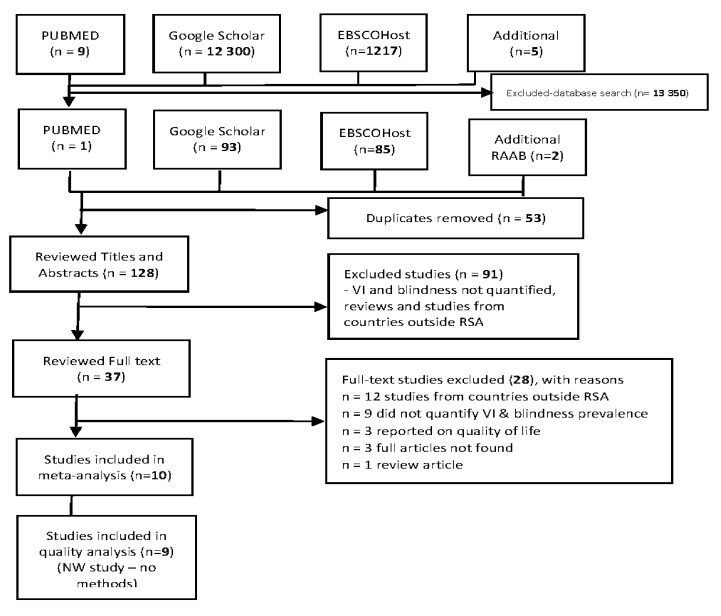
PRISMA chart showing the study selection process.

The selected studies, as outlined in the PRISMA chart (Figure 1), were stored in an EndNote X7 library, after which duplicates were removed. Thereafter, two independent co-screeners conducted title, abstract, and full article screening using standardized, piloted tools (Supplementary File S1). Where disputes arose, the reviewers discussed the articles to reach consensus. Finally, data extraction was conducted by the PI, followed by data charting using a tool developed, piloted, and finalized for this study (Supplementary File S2). Information was then extracted to give a quantitative account of the prevalence and major causes of VI and blindness in South Africa. 

***Collating, summarizing, and reporting results***

The extracted data were saved in an MS excel file (Supplementary Table S1) in preparation for the meta-analysis using MetaXL. The data were separated into two categories: visual impairment and blindness. Thereafter, major prevalences were analyzed. Ultimately, visual presentations and tables were used to present variations, distributions, and other significant parameters following analysis. 

### 2.2. Quality Appraisal

Quality appraisal is recommended as a means to confirm the lack of bias in study selection. In the present study, 9 of the 10 included articles were quality-indexed. This was performed by two independent reviewers, using the 2018 version of the Mixed-Method Appraisal Tool (MMAT) [20]. One study did not have a methodology section and, as such it was excluded from the quality assessment [21]. Since the latest tool does not grade studies, the scoring guidelines of MMAT 2011 [22] were adapted and used instead.

### 2.3. Data Analysis

An inverse variance heterogeneity (IVhet) model [23] in MetaXL was used to determine the Pooled prevalence estimates (PPE) and their 95% confidence intervals (CI) for the selected studies. This model was preferred because it maintains a correct coverage probability compared to the fixed-effect (FE) or random-effect (RE) models [23]. The PPE for each ocular problem was only determined where prevalence data were extracted from at least four studies. A graphical display of the prevalence estimates and their 95% CI was realized using Forest plots. To determine the degree of heterogeneity among the studies, the Cochran’s Q statistic and *I*^2^ were used, and the studies were categorized as having a low, moderate, and high degree of heterogeneity if the *I*^2^ was equal to 25%, 50%, and 75%, respectively. The Luis Furuya–Kanamori (LFK) index of the Doi plot and the funnel plot were used to assess publication bias [24]. For the doi plot, symmetry was evaluated using the LFK index, with indices values within ±1 regarded as having no asymmetry, indices exceeding ±1 but within ±2 regarded as having minor asymmetry, and those exceeding ±2 regarded as having major asymmetry and, thus, high publication bias [24]. 

## 3. Results

### 3.1. Characteristics of the Eligible Studies

The database search resulted in 13,527 studies. Another 4 studies were further found in the RAAB database, bringing the total to 13,531. Following various screening processes, 10 studies were included in the meta-analysis and 9 in the quality analysis. One study was omitted from the quality analysis as it did not include a methodology section [21]. Most of the studies were from KwaZulu-Natal (50%) [25,26,27,28,29], while those from the Western Cape and North West were 10% each [21,30]. The remaining 30% of the studies were conducted in the province of Limpopo [31,32,33]. Two studies were conducted on low-vision patients and showed a high prevalence of oculocutaneous albinism (OCA) as a cause of URE and MSVI [26,29]. Other lesser causes of MSVI were macular degeneration, complications following cataract surgery, and “other” causes. The quality of all the included studies was above average, with reviewers individually scoring studies from 60% to 100% (Supplementary File S3) with an an overall average score of 71.11%. According to the independent reviewers, all the included studies were of relatively good quality. From the total sample of 13,525 participants, approximately 28% were males, with the remaining 72% being female.

### 3.2. Pooling Prevalence Estimates and Heterogeneity Analyses

**Figure 2:**
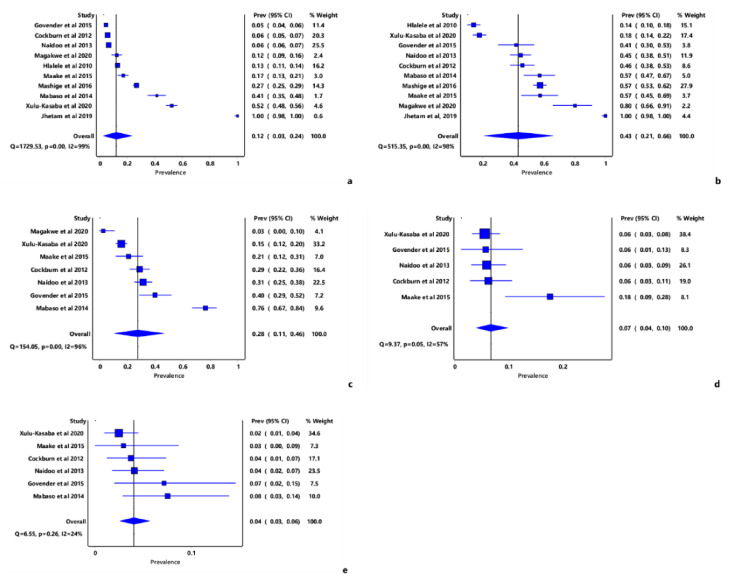
Forest plots of the prevalence estimates of MSVI and its major causes in South Africa; (**a**) Overall MSVI prevalence, (**b**) URE, (**c**) Cataract, (**d**) Glaucoma, (**e**) diabetic retinopathy.

The overall pooled prevalence estimate (PPE) of moderate to severe visual impairment (MSVI) was 12% (IVhet: 95% CI: 3.0–24.0) (Figure 2a). Among the major causes of moderate to severe visual impairment (MSVI), uncorrected refractive error (URE) had the PPE of 43% (95% CI: 21.0–66.0) (Figure 2b). The least cause of MSVI according to our results were diabetic retinopathy (DR), which had a PPE of 4% (95% CI: 3.0–6.0). Other causes of MSVI were cataracts and glaucoma (Figure 2c,d). 

**Figure 3:**
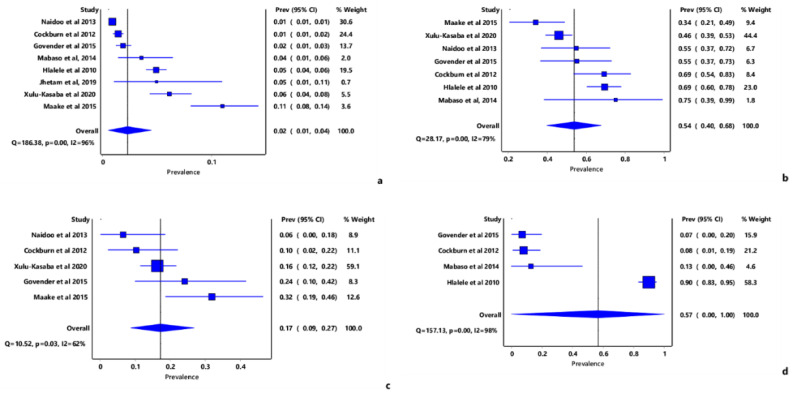
Forest plot of the prevalence estimates of blindness and its major causes in South Africa; (**a**) Overall blindness prevalence, (**b**) Cataract, (**c**) Glaucoma, (**d**) diabetic retinopathy.

The overall pooled prevalence estimate of blindness was 2% (IVhet: 95% CI: 1.0–4.0) (Figure 3a and Supplementary File S4). The major causes of blindness in the included studies were diabetic retinopathy, which had the PPE of 57% (IVhet: 95% CI: 0.0–100) (Figure 3b), and cataracts with a prevalence of 54% (IVhet: 95% CI: 40.0–68.0). Glaucoma was the least cause of blindness, although its prevalence was also high (17%, IVhet: 95% CI: 9.0–27.0). The high level of heterogeneity measured as *I*^2^ in most pooled estimates ranged from 24 to 98. Furthermore, assessment of the funnel plot and doi plot ruled out a significant (Supplementary Files S5 and S6) publication bias. 

## 4. Discussion

Ocular health and good vision form a vital part of a healthy, independent, and well-functioning individual. Good vision and satisfactory ocular health allow one to become educated in any discipline, become employable, enjoy a satisfactory quality of life, and earn a living [34,35,36,37]. Furthermore, the absence of blindness and visual impairment improves an individual’s choices in nutrition and hygiene [38,39]. The results from the current study show that visual impairment (VI) remains a major public health challenge in South Africa. Visual impairment is one of many economic and health factors linked to an ageing global population [40,41,42,43]. 

According to the WHO, the number of people with visual impairment has increased by 450% in four years, from 285 million in 2014 [44] to 1.3 billion in 2018; impacting significantly on global economies [45]. This increase was attributed to causes that differed in high-income and low-income countries [46,47,48]. Our study suggests that the leading cause of VI was URE, while diabetic retinopathy (DR) was the least cause of VI. These findings corroborate observations made within Sub-Sahara Africa that URE is the leading cause of visual impairment [47,49,50,51]. Other causes of visual impairment observed in our study, as well as in other studies, were untreated cataracts and glaucoma. Observations from high-income countries suggest that diabetic retinopathy, glaucoma, age-related macular degeneration, and retinopathy of prematurity are the major causes of VI [50,52,53]. This is most probably due to a lifestyle characterized by fast foods consumption and other habits of convenience in high-income countries. Age-related VI is most probably due to the fact that people live longer in those areas due to a relatively good and accessible primary health care. Interestingly, our study reported a low prevalence of trachoma, found only in one province [21]. This low prevalence might be a reflection of the improved social infrastructure in South Africa where clean drinking water and sanitation are accessible to over 83% of the population [54]. Trachoma, the leading cause of infectious and avoidable blindness globally, is caused by an intracellular bacterium and is most commonly found in young children [55]. It is largely spread through poor hygiene practices resulting from a lack of clean, running water; as such, some authors have identified it as a disease of poverty [56,57]. While it remains a major cause for concern in LMICs, Kenya, Ethiopia, and the East and West regions of SSA [58,59,60], it is pleasing to know that in 2021, remarkable progress had been made towards eradicating this endemic disease in the African continent [61,62]. 

As most VI is largely manageable and avoidable [46], it is concerning that it is the leading cause of disability in South Africa [13]. Spectacles are the simplest method to manage most URE, [63] following examination by an optometrist; hence, the prevalence of URE should be improved in this country if it has an adequate supply of optometrists, suitably distributed for accessibility to the greater population. 

Our study observed that cataracts were the second major cause of VI and were also the second leading cause of blindness. However, previous studies in other LMICs have attributed most blindness to cataracts [64,65,66,67,68,69,70], showing the need for interventions into improving cataract surgery rates (CSR) on the continent. The similarity of our study to those regarding many SSA countries is that the causes of VI and blindness are probably due to a lack of resources and early intervention, caused by low staff availability. Nonetheless, a study conducted by Aboobaker et al. [71] found accessibility, awareness, and affordability as the main barriers to cataract surgery in Africa. In South Africa, Lecuona et al. [70] cited the shortage in Human Resources for eye Health (HReH) as the greatest reason for high volumes of unmet surgery rates. Others have added planning, health systems, and continuous training as factors responsible for the lagging CSR, which is said to be worse in South Africa than in other developing countries [72,73,74,75,76]. The district health model, used in South Africa, prescribes that the first level of treatment should be at the primary health level, from where patients need to be referred to a secondary, then tertiary health facility if needed [77]. In most cases, the staff at the PHC facilities are not well trained in eye health [78,79,80], resulting in triage not being accurately managed at the initial visit. This, coupled with overloaded inter-hospital shuttle services, understaffing, and a shortage of resources, results in bottlenecking within the entire system, causing backlog and delays in procedures and surgeries. It is evident here that CSR needs to be prioritized and improved to manage this prevalent cause of avoidable blindness in the country. 

Diabetic retinopathy and glaucoma have also emerged as concerning prevalent major causes of visual impairment and blindness in the whole SSA, and this is also true in South Africa. As the leading cause of blindness amongst working adults, diabetic retinopathy should be managed better through early screening and detection. Likewise, glaucoma is also easy to detect and manage, especially when identified early. Many studies have recommended screening at a primary health level where management can be facilitated with the use of teleophthalmology [81,82,83]. Glaucoma studies have also shown that early intervention at the primary health level can be useful in preventing blindness later on in life [84,85]. In these studies, the interpretation of disease progression was done by ophthalmologists, retinal specialists, and optometrists, and triage and management were then facilitated at the primary health level. To alleviate further avoidable blindness, it would be advisable for South Africa to consider these interventions to manage disease progression and seek to eliminate blindness and visual impairment due to glaucoma and diabetic retinopathy in the future.

Of note is the fact that trachoma was only mentioned in the North West province. This “disease of poverty”, known as the leading blindness-causing infectious disease, has been eradicated in most resourced economies like Europe and the Western World [56]. It is concerning that this disease, spread through poor sanitation, is still found in South Africa. Policymakers will need to look into the reasons for trachoma’s prevalence in the North West province and seek to find ways to work with other departments to improve sanitation and basic infrastructure to eliminate this disease.

### Limitations and Areas for Future Research

More than 50% of the included studies came from KwaZulu-Natal, one of the nine provinces of South Africa, a factor that may have possibly skewed the findings of the study. The demographics and population types in KwaZulu-Natal are rather different from those of areas like the Western Cape [86]. The province of KwaZulu-Natal has a far greater Indian population than that of the Western Cape, for example. Factors related to this may influence their susceptibility to certain conditions, thus somewhat influencing the results. In future, more population-based studies should be conducted in provinces such as the Eastern Cape, which has the largest poverty-stricken population in South Africa [87], and Gauteng, the most densely populated province in the country [88]. Future research should also explore the availability of Human Resources for Eye Health, in addition to studies that have assessed this in the country [89], to explore the extent to which VI and blindness can be managed in the country.

## 5. Conclusions

The disease profile of the major causes of VI and blindness in South Africa has been identified. URE is the leading cause of MSVI, followed by cataracts, glaucoma, and diabetic retinopathy. Blindness in South Africa is largely due to cataracts, glaucoma, and diabetic retinopathy. These are spread around the country, with a greater presence in KwaZulu-Natal, where most studies were conducted. It is important to address these conditions by enabling HReH frameworks and systems that will seek to alleviate these causes of unnecessary blindness in this country.

## Figures and Tables

**Table 1:** PCC framework.

Population	People with visual anomalies and ocular disease
Concept	Prevalence of disease The leading global causes of visual impairment and blindness were used to identify studies—uncorrected refractive errors (URE), cataract, macular degeneration, glaucoma, diabetic retinopathy, corneal opacity, and trachoma.
Context	South Africa in the period 2010–2020, as this was part of a larger study that explored prevalences of VI and blindness in Sub-Saharan Africa (SSA) in the same period.

## Data Availability

All information used in this review is already available in the public domain.

## Supplementary Materials

The following are available online at https://www.mdpi.com/article/10.3390/tropicalmed7020034/s1, Supplementary File S1: Screening tool, Supplementary File S2: Data charting Tool, Supplementary File S3: Quality Index Scores, Supplementary Table S1: Data set used for analysis, Supplementary File S4-1: Blindness cataracts forest plot, Supplementary File S4-2: Blindness DR forest plot, Supplementary File S4-3: Blindness forest plot, Supplementary File S4-4: Blindness Glaucoma forest plot, Supplementary File S4-5: Cataracts forest plot, Supplementary File S4-6: MSVI DR forest plot, Supplementary File S4-7: MSVI prev forest plot, Supplementary File S4-8: MSVI Glaucoma forest plot, Supplementary File S4-9: MSVI URE forest plot, Supplementary File S5-1: Funnel Plot Blindness DR, Supplementary File S5-2: Funnel plot Blindness Glaucoma, Supplementary File S5-3: Funnel plot blindness, Supplementary File S5-4: Funnel plot cataracts Blindness, Supplementary File S5-5: MSVI cataracts funnel plot, Supplementary File S5-6: MSVI Dr funnel plot, Supplementary File S5-7: MSVI Galucoma funnel plot, Supplementary File S5-8: MSVI prev funnel plot, Supplementary File S5-9: MSVI URE funnel plot, Supplementary File S6-1: Doi plot blindness cataracts, Supplementary File S6-2: Doi plot Blindness DR, Supplementary File S6-3: Doi plot Blindness, Supplementary File S6-4: Doi plots Blindness glaucoma, Supplementary File S6-5: MSVI cataracts doi plot, Supplementary File S6-6: MSVI DR doi plot, Supplementary File S6-7: MSVI Glaucoma doi plot, Supplementary File S6-8: MSVI Prev doi plot, Supplementary File S6-9: MSVI URE doi plot.
