# Supplementary material for: Prevalence of the Burden of Diseases Causing Visual Impairment and Blindness in South Africa in the Period 2010–2020: A Systematic Scoping Review and Meta-Analysis
Source: Trop Med Infect Dis. 2022 Feb 21;7(2):34. doi: 10.3390/tropicalmed7020034 (PMC8877290; doi:10.3390/tropicalmed7020034)
Supplement: Supplementary file 1 [file tropicalmed-07-00034-s001.zip › Supplementary 1,2,3/Supplementary File 1 - Screening tool.pdf]

A scoping review on major causes of VI in RSA- final Abstract Screening

ABSTRACT SCREENING

1. AUTHOR & DATE

____
____
____
____
____

2. TITLE

____
____
____
____
____

3. Was the study done on human beings?

Mark only one oval.

- ⬭ YES
- ⬭ NO

4. [illegible]

4. Does the study show evidence on the presence of visual impairment/refractive error/ocular conditions causing VI, or any of the related synonyms and MeSH terms (see list provided) in a certain population?

Mark only one oval.

- YES
- NO

5. Was the study carried out in South Africa?

Mark only one oval.

- YES
- NO

6. Was the study carried out in the period 2010 - 2020?

Mark only one oval.

- YES
- NO

7. SCREENER INITIALS

____

This content is neither created nor endorsed by Google.

Google Forms
