# Supplementary material for: Prevalence of the Burden of Diseases Causing Visual Impairment and Blindness in South Africa in the Period 2010–2020: A Systematic Scoping Review and Meta-Analysis
Source: Trop Med Infect Dis. 2022 Feb 21;7(2):34. doi: 10.3390/tropicalmed7020034 (PMC8877290; doi:10.3390/tropicalmed7020034)
Supplement: Supplementary file 1 [file tropicalmed-07-00034-s001.zip › Supplementary 1,2,3/Supplementary File 2 - Data charting Tool.pdf]

# Data extraction - VI and Blindness in RSA

FINAL VERSION - VI & BLINDNESS

* Required

1. Author/Main PI and Year of Study - write only first author et al. *

2. Title of Study *

3. Total sample size *

4. Total nr of MALES *

5. Total nr of FEMALES *

6. Number of blind people (VA < 3/60) *

7. Number of VI people (MSVI) *

8. Blind MALES over age 50 *

9. MSVI males over age 50 *

10. Blind FEMALES over age 50 *

11. MSVI females over age 50 *

12. Blind due to CATARACTS *

13. MSVI due to CATARACTS *

14. Blind due to REFRACTIVE ERROR *

15. MSVI due to REFRACTIVE ERROR *

16. Blind due to DIABETIC RETINOPATHY *

17. MSVI due to DIABETIC RETINOPATHY *

18. Blind due to GLAUCOMA *

19. MSVI due to GLAUCOMA *

20. BLIND due to TRACHOMA *

21. MSVI due to TRACHOMA

22. BLIND due to MACULAR DEGENERATION *

23. MSVI due to MACULAR DEGENERATION *

24. BLIND due to ONCHOCERCIASIS *

25. MSVI due to ONCHOCERCIASIS *

*Example: January 7, 2019*

26. BLIND due to TRACHOMA *

27. MSVI due to TRACHOMA *

28. BLIND due to OTHER CAUSES not mentioned above *

29. MSVI due to OTHER CAUSES not mentioned above *

30. Data capturer - initials *

This content is neither created nor endorsed by Google.

Google Forms
