# Supplementary material for: Prevalence of the Burden of Diseases Causing Visual Impairment and Blindness in South Africa in the Period 2010–2020: A Systematic Scoping Review and Meta-Analysis
Source: Trop Med Infect Dis. 2022 Feb 21;7(2):34. doi: 10.3390/tropicalmed7020034 (PMC8877290; doi:10.3390/tropicalmed7020034)
Supplement: Supplementary file 1 [file tropicalmed-07-00034-s001.zip › Supplementary 1,2,3/Supplementary Table S1 Data set used for analysis final.pdf]

MSVI

AUTHOR & DATE	TITLE	PROVINC	TOTAL SAMPLE	MALES	FEMALE	MSVI	MSVI - Fem 50-	MSVI - Males 50+	Cataract MSVI	Refr Error MSVI	Macular d degen	Glaucoma
Cockburn et al 2012	Prevalence, causes and soc	WC	2750	1037	1713	160			46	73	4	10
Govender et al 2015	Rapid assessment of avoida	KZN	1542	463	1079	70	53	17	28	29		4
Hlalele et al 2010	Rapid Assessment of Vision	NW	2197			281						
Jhetam et al, 2019	Ocular findings and vision st	KZN	81	33	48	81						
Maake et al 2015	Prevalence and causes of vi	LP	400	161	239	68			14	39		12
Mabaso	Risk factors for visual impair	LP	225	161	64	93			71	53		
Magakwe et al 2020	Prevalence and Distribution	LP	326	161	165	40			1	32		
Mashige et al 2016	Prevalence of refractive erro	KZN	1939	483	1456	518				297		
Naidoo et al 2013	A population-based study of	KZN	3444	1464	1980	220			69	98		119
Xulu-Kasaba et al 2020	A profile of patients presenti	KZN	621	280	341	324	163	133	50	57	42	19
			13525	3780	6006							

BLINDNESS PREV

AUTHOR & DATE	TITLE	PROVINCE	Blind	Blind Fem 50-	Blind Males 50	Cataract Blind	Refr Err Blind	Corn opac Blind	Macular d Blind	Glaucoma	DR- Blind	Corneal sca issues
Cockburn et al 2012	Prevalence, causes and soc	CT	39	3	2	27	0	3	4	4	3	3
Govender et al 2015	Rapid assessment of avoida	KZN	29			16	2	1		7	2	1
Hlalele et al 2010	Rapid Assessment of Vision	NW	108			75					97	
Jhetam et al, 2019	Ocular findings and vision st	KZN	4	0	0							
Maake et al 2015	Prevalence and causes of vi	LP	44			15				14		8
Mabaso et al, 2014	Risk factors for visual impair	LP	8			6					1	
Naidoo et al 2013	A population-based study of	KZN	31			17	4			2		

Xulu-Kasaba et al 2020	Rapid assessment of refract	KZN	209			96				34		6

DR- MSVI	Corneal issues	Other causes	cat surg cor MSVI	HPT ret.	albinism	other
6	2	31	2			
5		3	1			
					81	
2	30	3				
7						
	3					
81	10			9		
8	39			9	141	113

Other cau post seg	Phthisis Blind	Cat surg complic.	Aphakia	Trachoma	optic atroph	Quality assess
13	1	0	1			60%
		0				70%
				3		none
						60%
						70%
						70%
						70%

					38	80%

EAST AI	SOUTHE	WEST AFRICA
Mashayo et al 2015		Muhammad et al 2016
