# Supplementary figures and images for: Prevalence of the Burden of Diseases Causing Visual Impairment and Blindness in South Africa in the Period 2010–2020: A Systematic Scoping Review and Meta-Analysis
Source: Trop Med Infect Dis. 2022 Feb 21;7(2):34. doi: 10.3390/tropicalmed7020034 (PMC8877290; doi:10.3390/tropicalmed7020034)

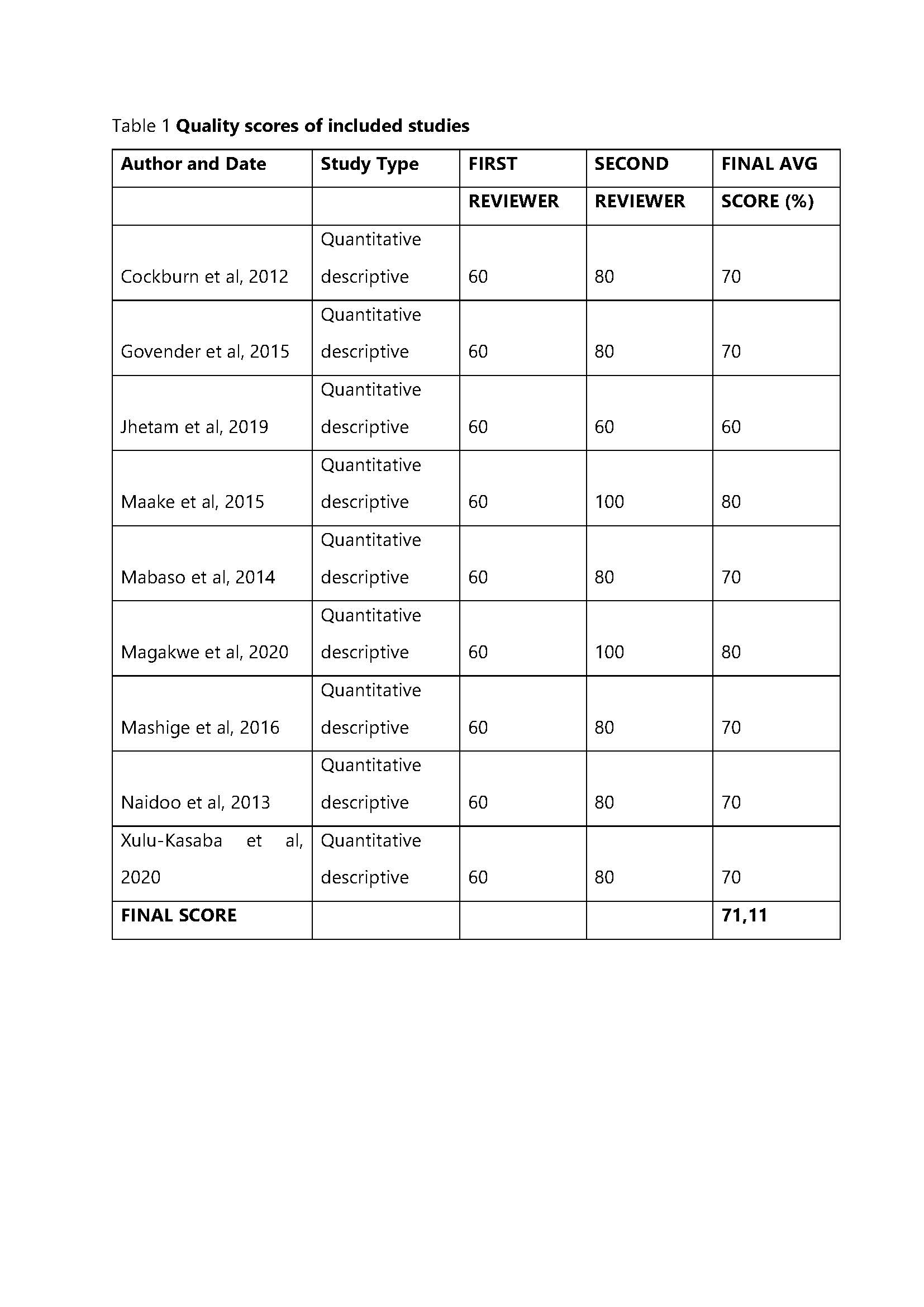

Supplement: Supplementary file 1 [file tropicalmed-07-00034-s001.zip › Supplementary 1,2,3/Supplementary File 3 - Quality Index Scores.jpg]

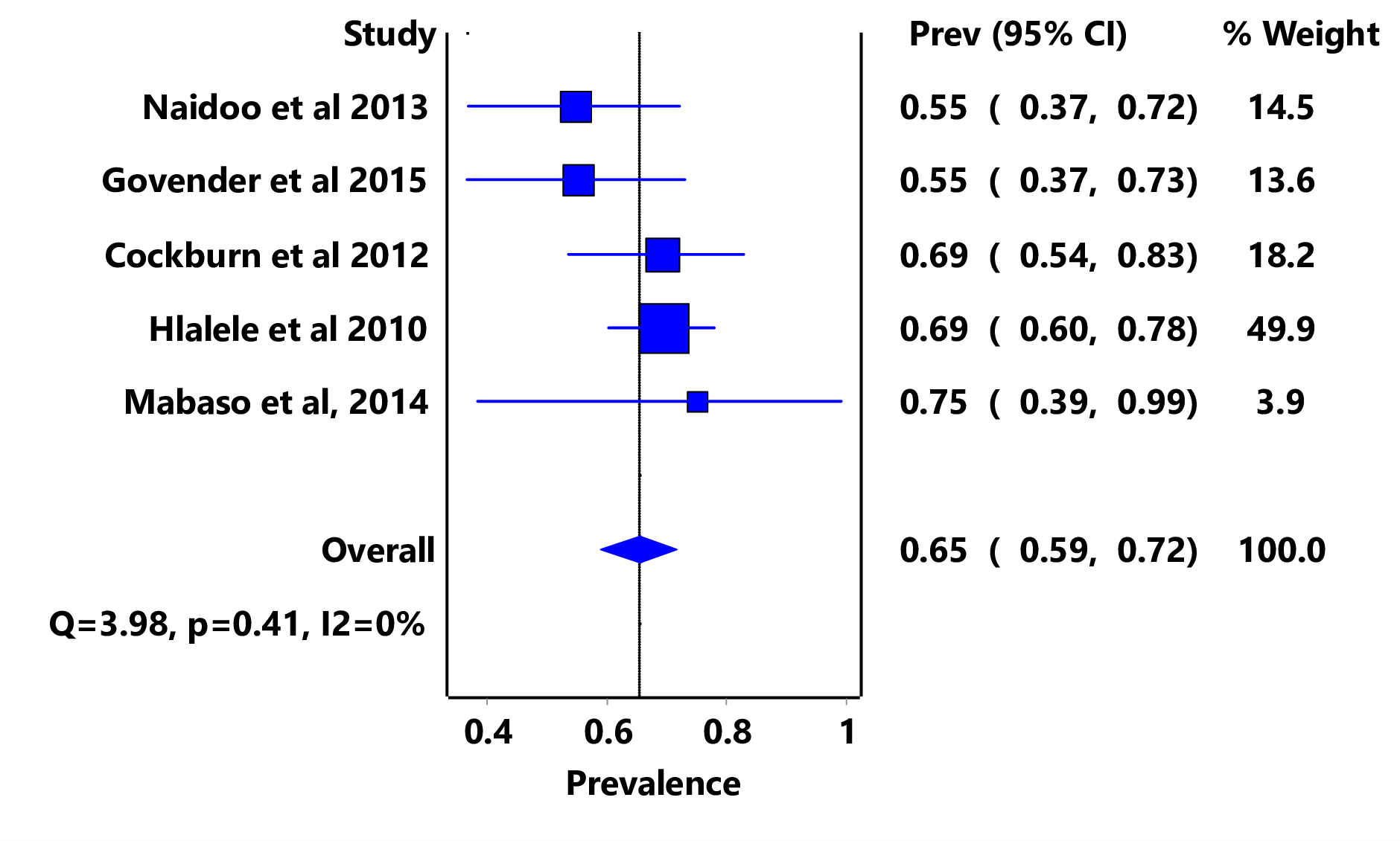

Supplement: Supplementary file 1 [file tropicalmed-07-00034-s001.zip › Supplementary File 4,5,6/Supplementary File 4-1 Blindness cataracts forest plot.tif]

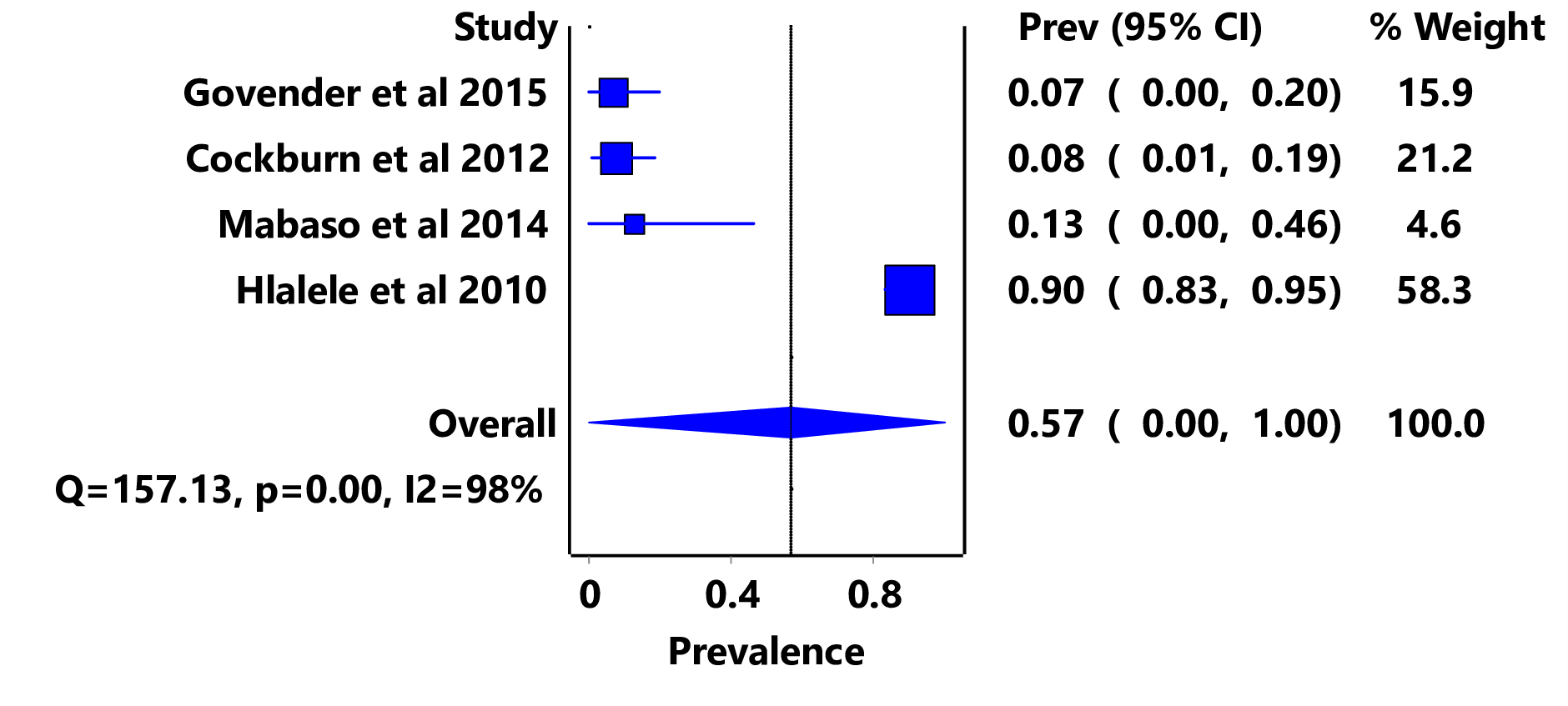

Supplement: Supplementary file 1 [file tropicalmed-07-00034-s001.zip › Supplementary File 4,5,6/Supplementary File 4-2 Blindness DR forest plot.tif]

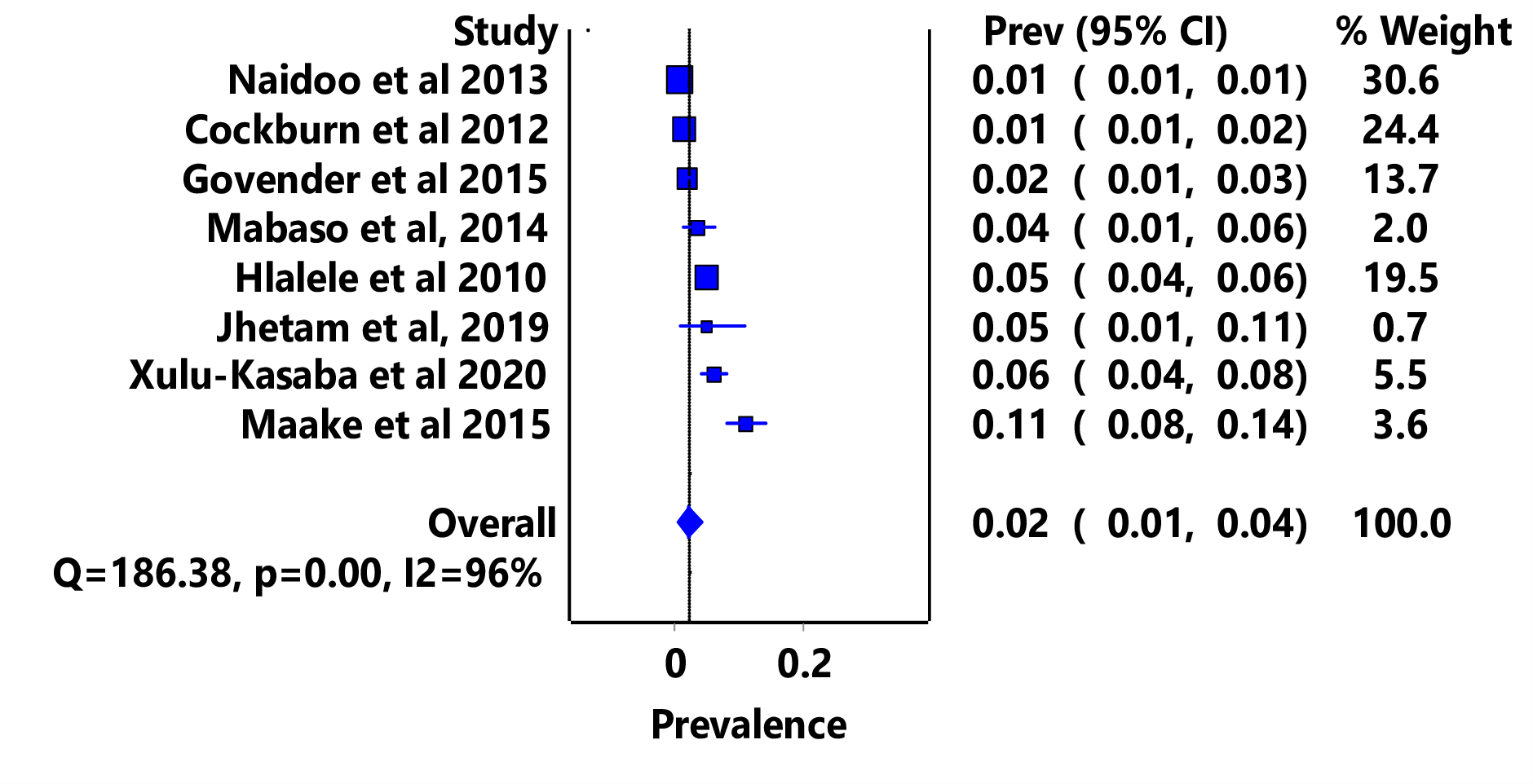

Supplement: Supplementary file 1 [file tropicalmed-07-00034-s001.zip › Supplementary File 4,5,6/Supplementary File 4-3 Blindness forest plot.tif]

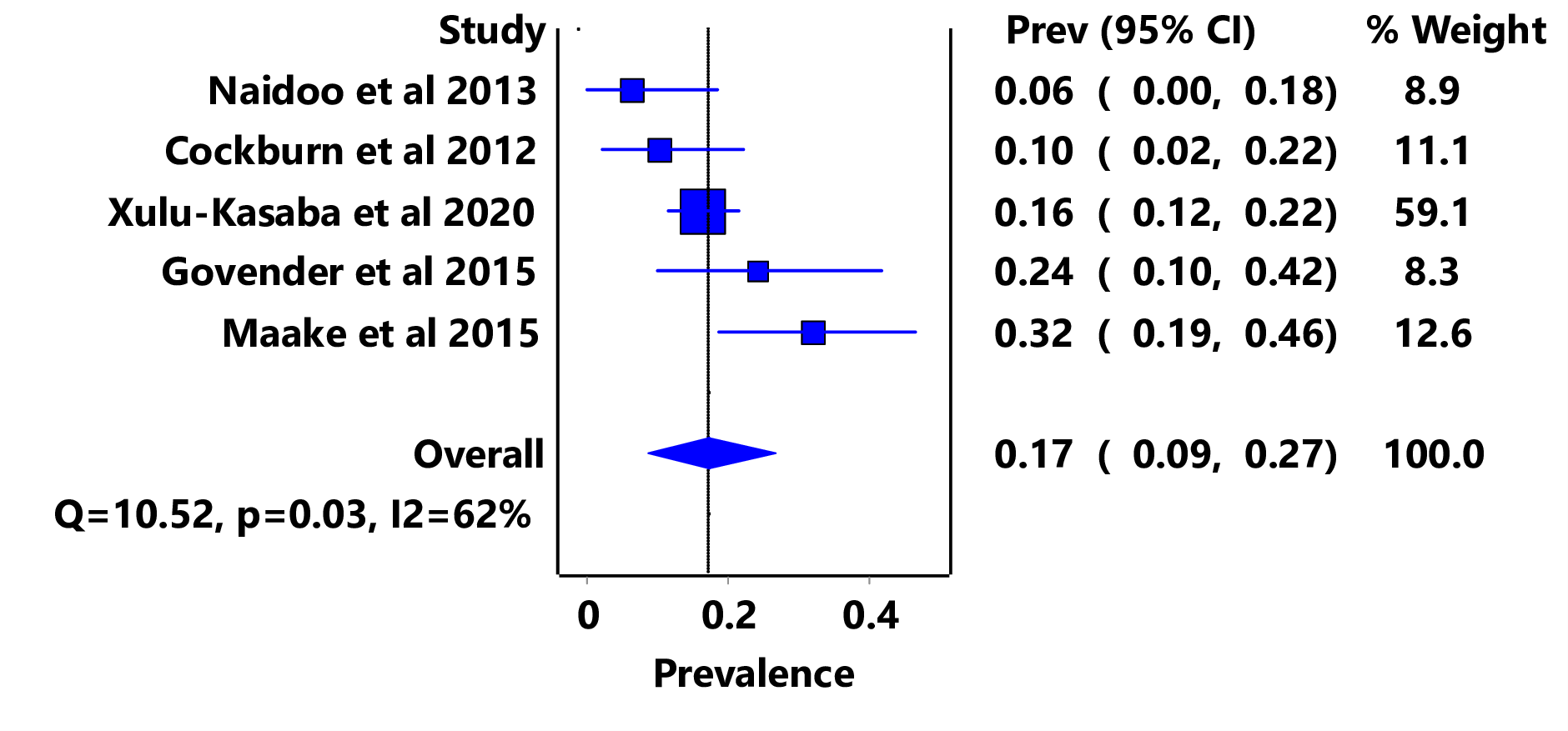

Supplement: Supplementary file 1 [file tropicalmed-07-00034-s001.zip › Supplementary File 4,5,6/Supplementary File 4-4 Blindness Glaucoma forest plot.tif]

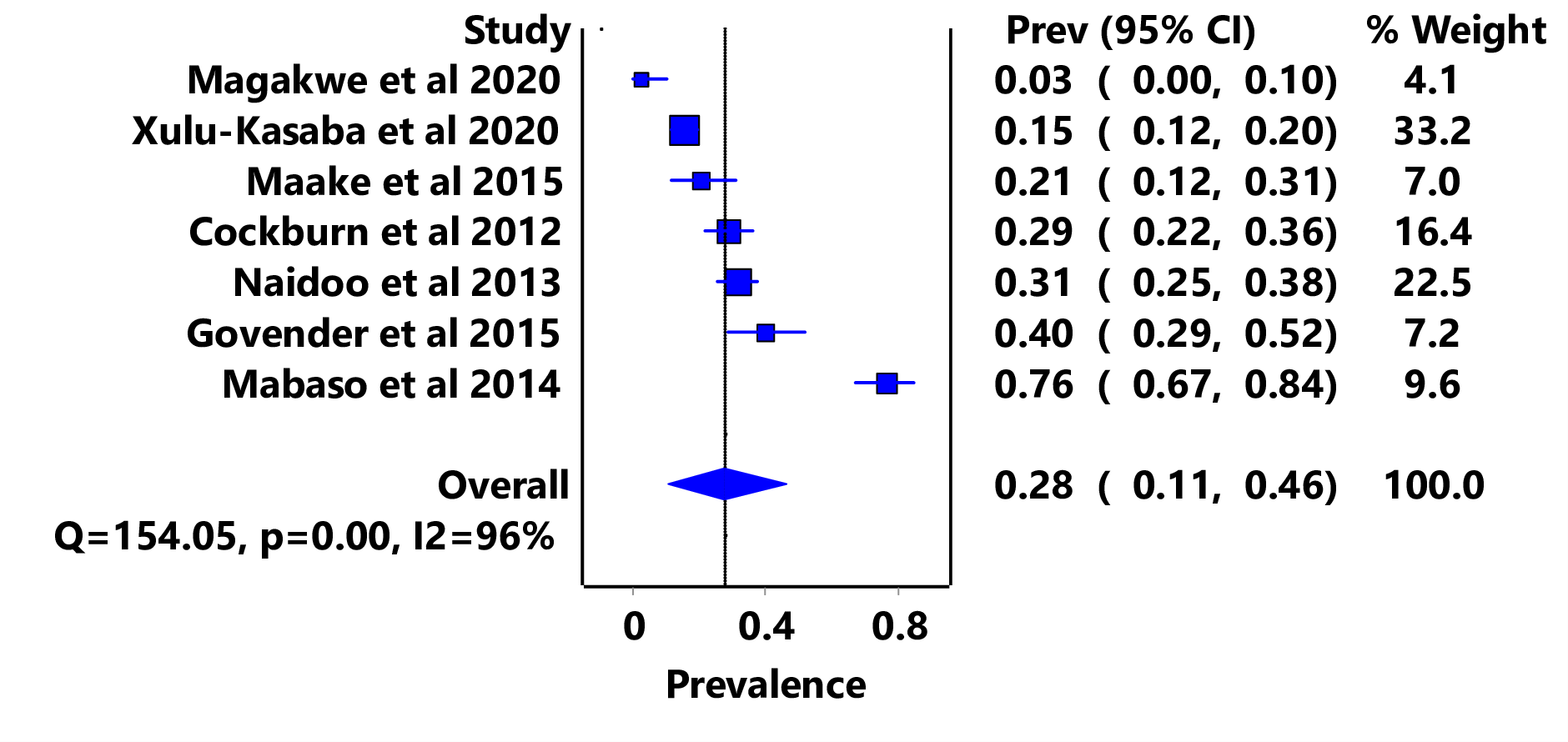

Supplement: Supplementary file 1 [file tropicalmed-07-00034-s001.zip › Supplementary File 4,5,6/Supplementary File 4-5 Cataracts forest plot.tif]

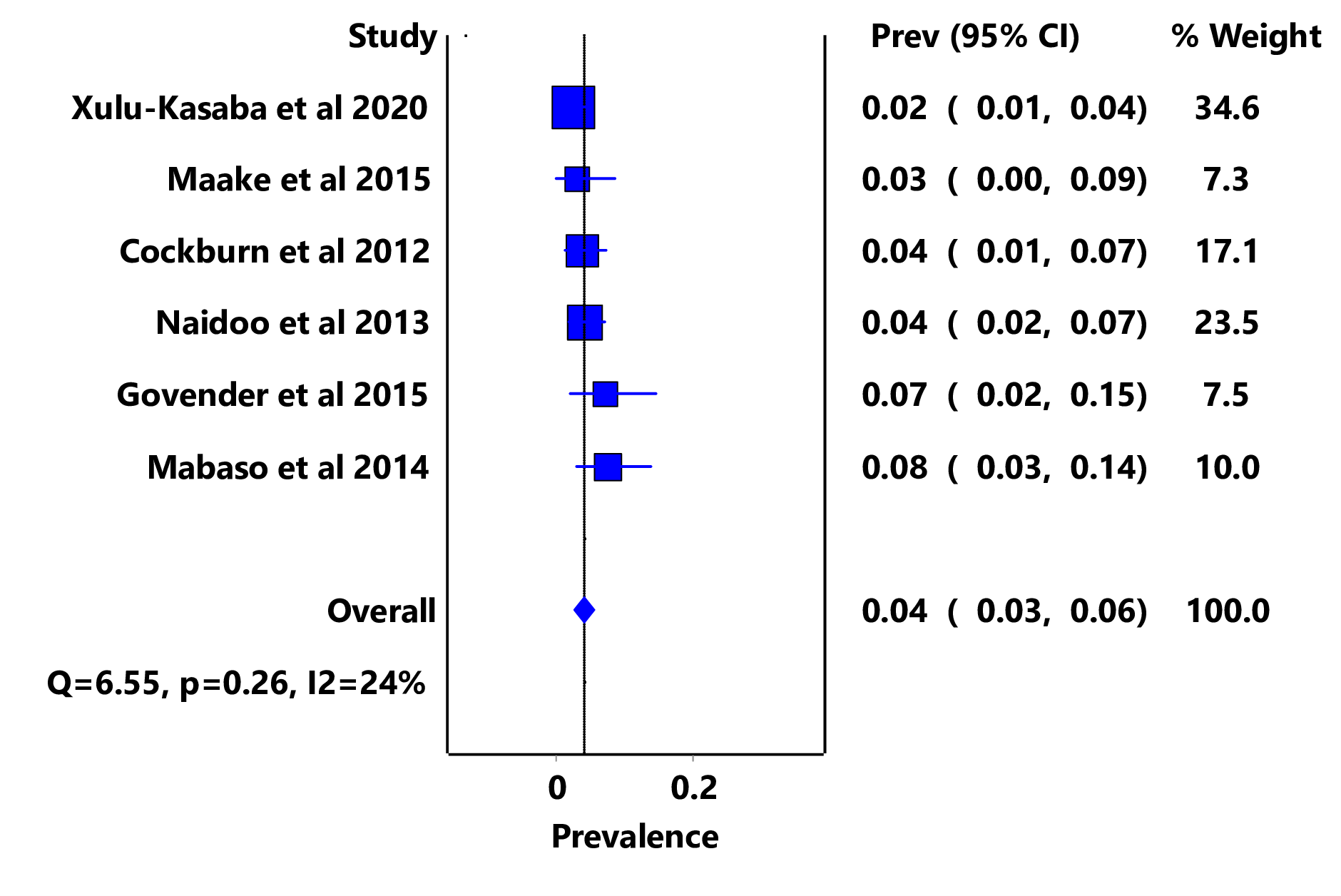

Supplement: Supplementary file 1 [file tropicalmed-07-00034-s001.zip › Supplementary File 4,5,6/Supplementary File 4-6 MSVI DR forest plot.tif]

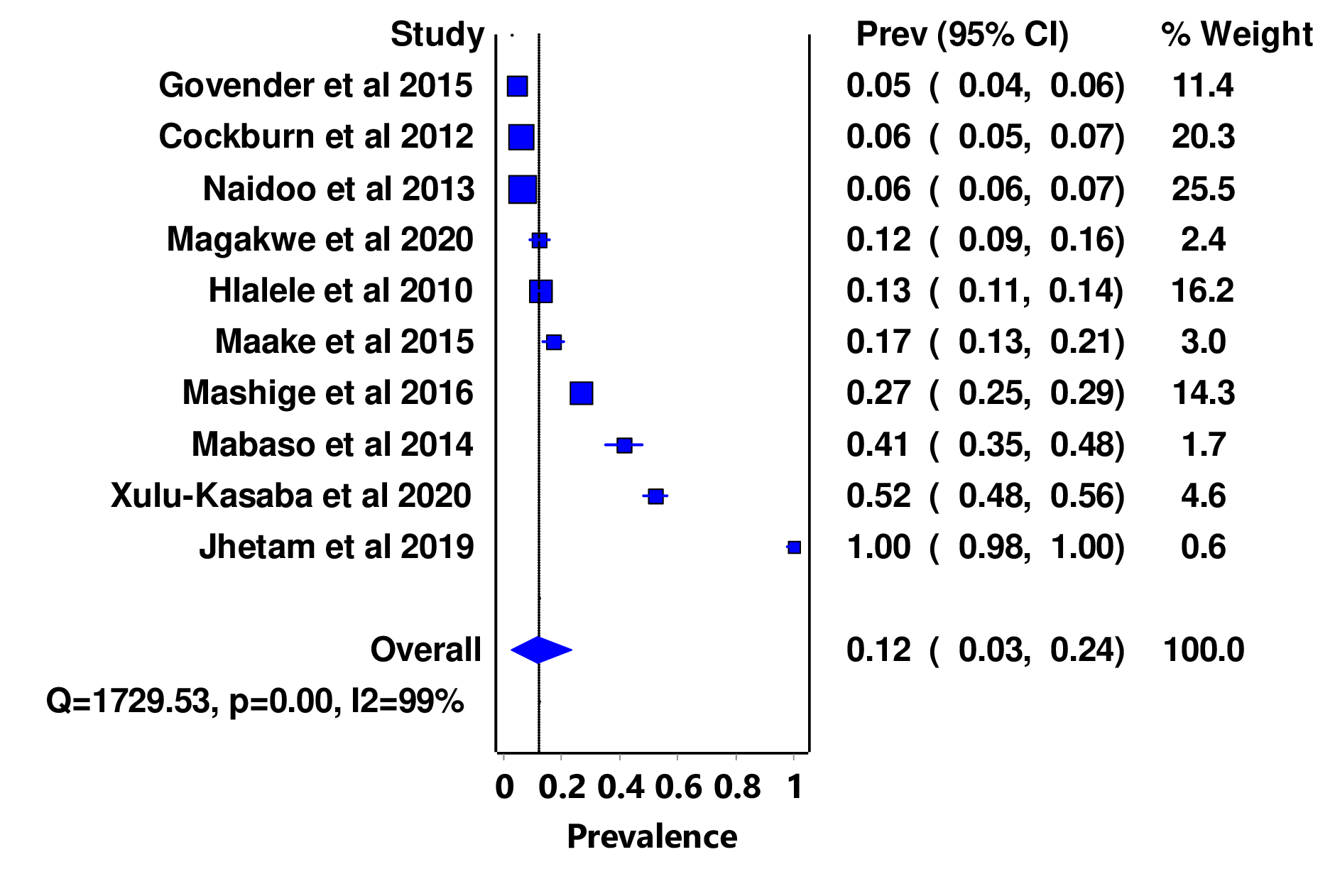

Supplement: Supplementary file 1 [file tropicalmed-07-00034-s001.zip › Supplementary File 4,5,6/Supplementary File 4-7 MSVI prev forest plot.tif]

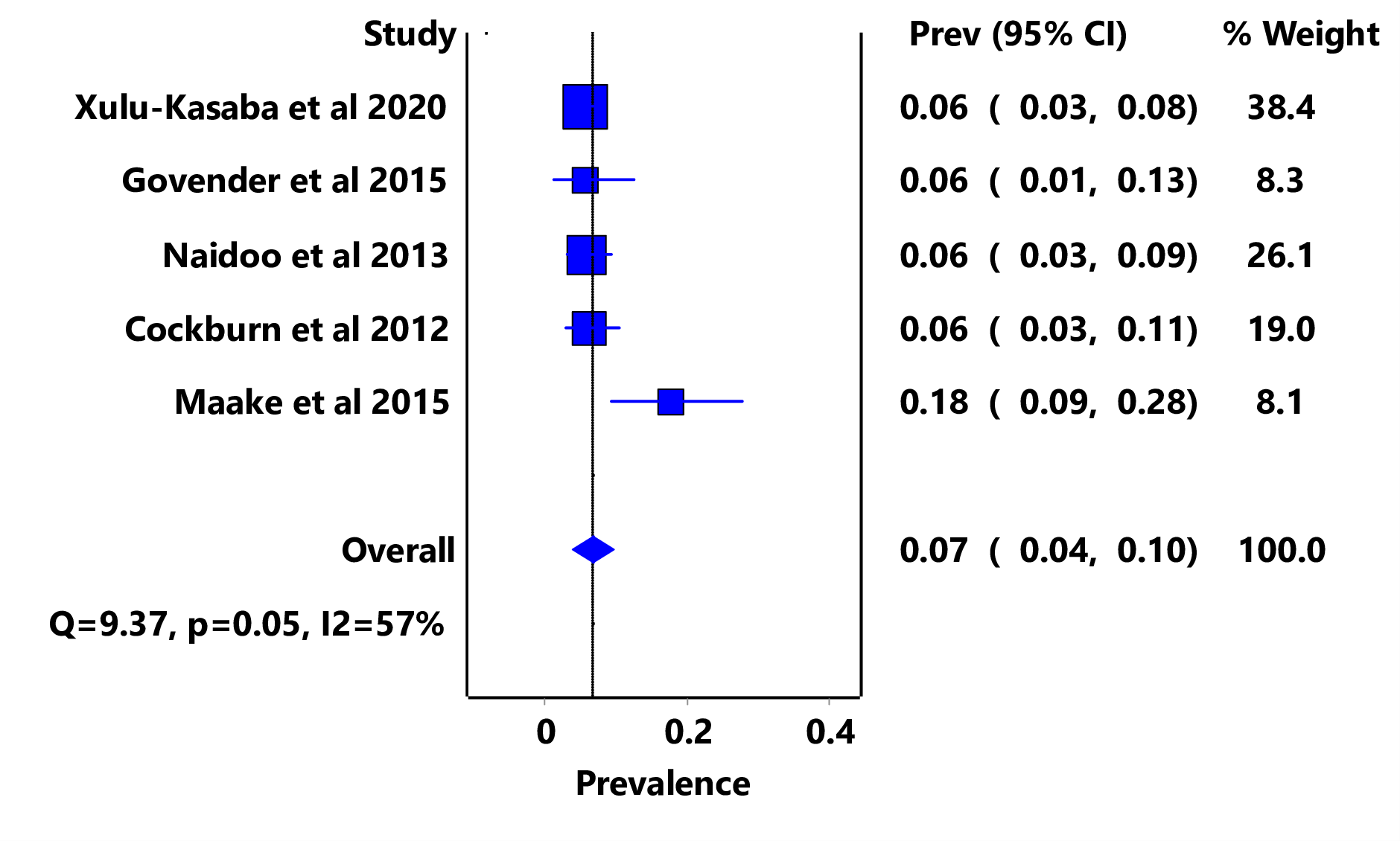

Supplement: Supplementary file 1 [file tropicalmed-07-00034-s001.zip › Supplementary File 4,5,6/Supplementary File 4-8 MSVI Glaucoma forest plot.tif]

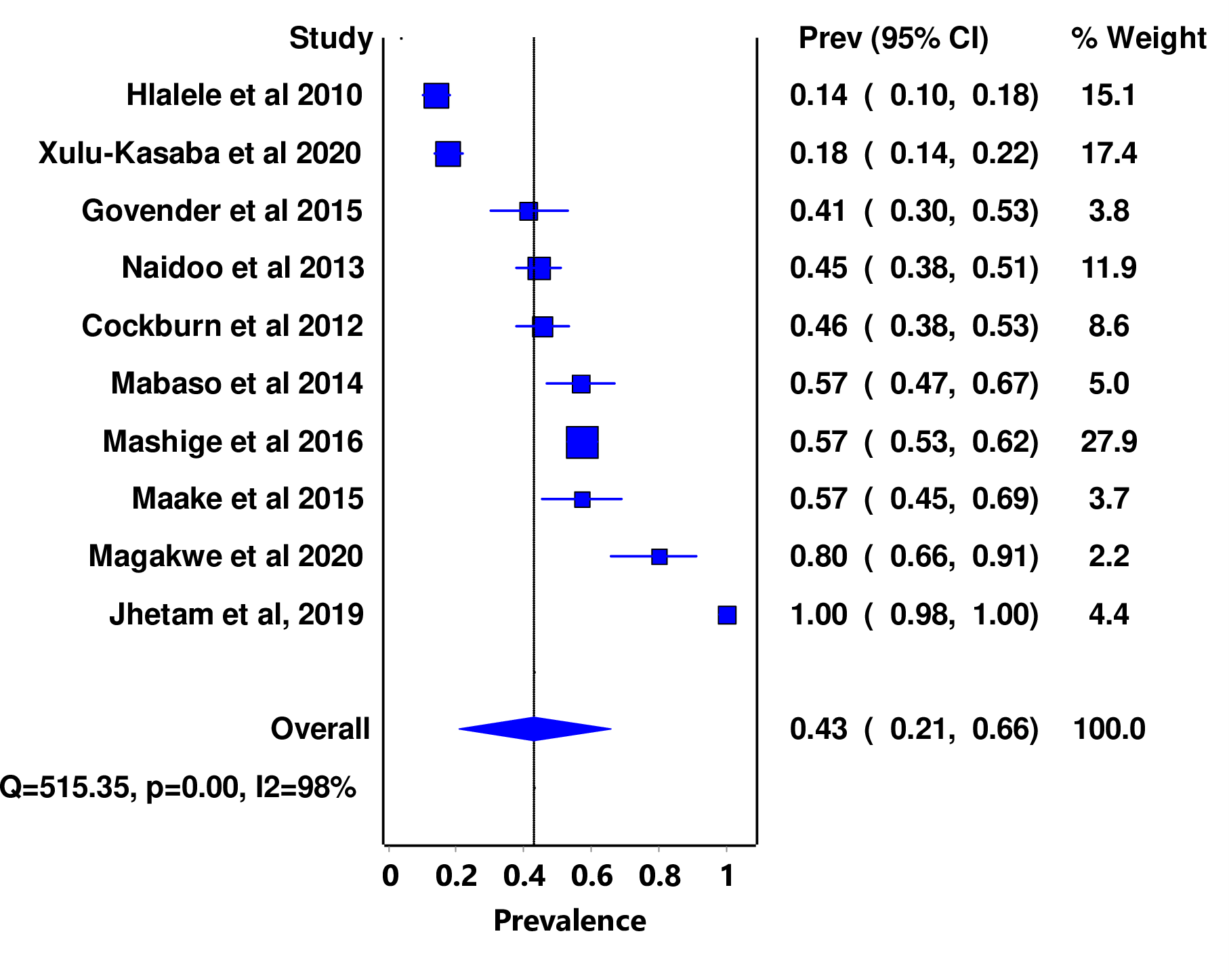

Supplement: Supplementary file 1 [file tropicalmed-07-00034-s001.zip › Supplementary File 4,5,6/Supplementary File 4-9 MSVI URE forest plot.tif]

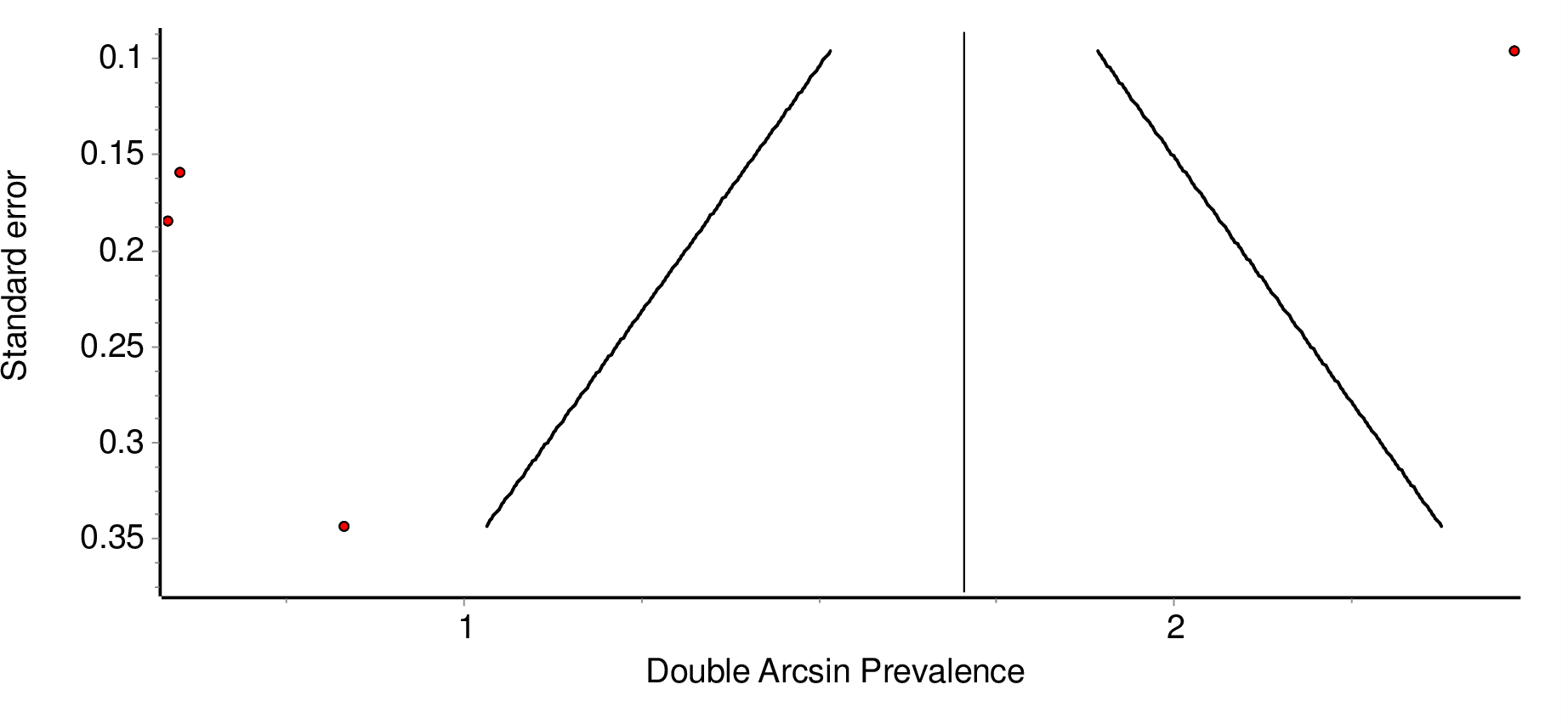

Supplement: Supplementary file 1 [file tropicalmed-07-00034-s001.zip › Supplementary File 4,5,6/Supplementary File 5-1 Funnel Plot Blindness DR.tif]

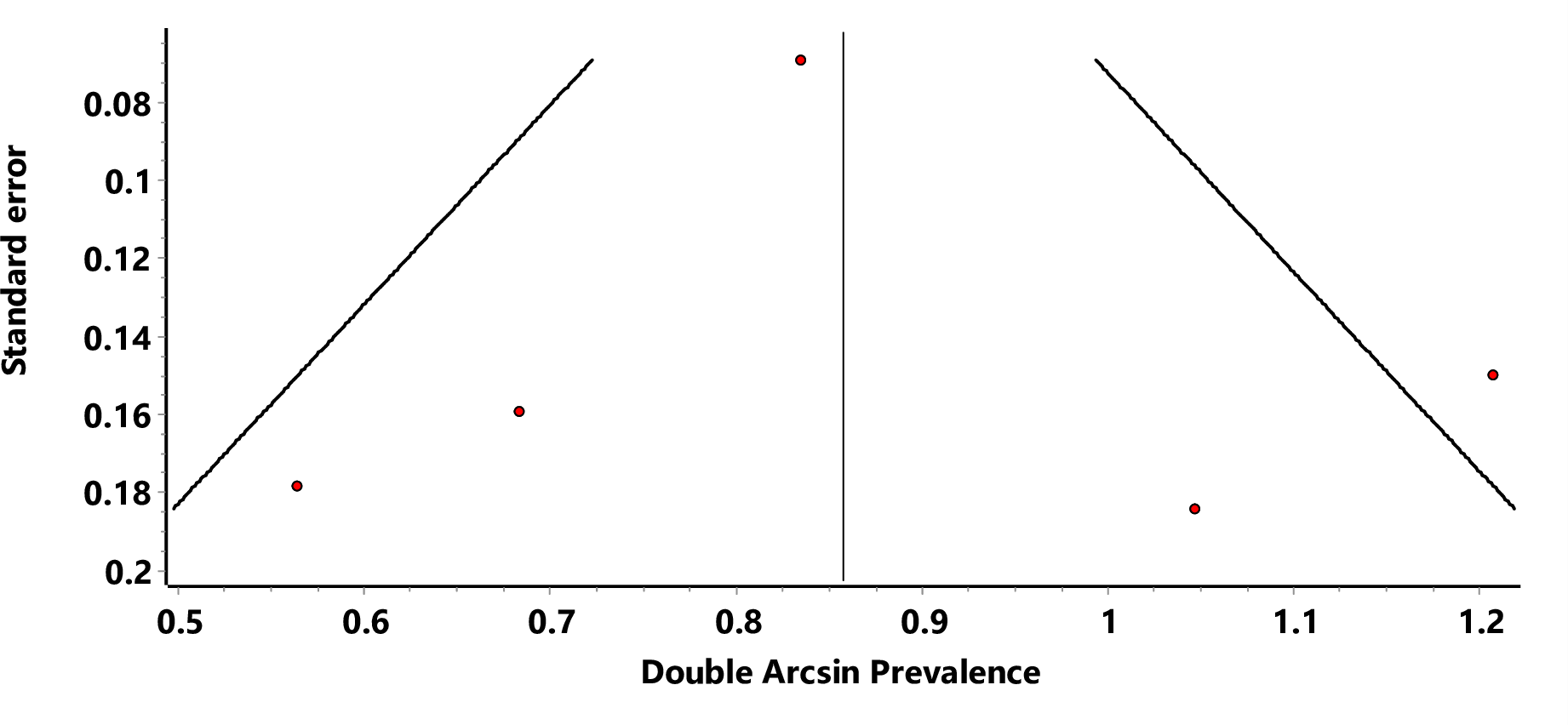

Supplement: Supplementary file 1 [file tropicalmed-07-00034-s001.zip › Supplementary File 4,5,6/Supplementary File 5-2 Funnel plot Blindness Glaucoma.tif]

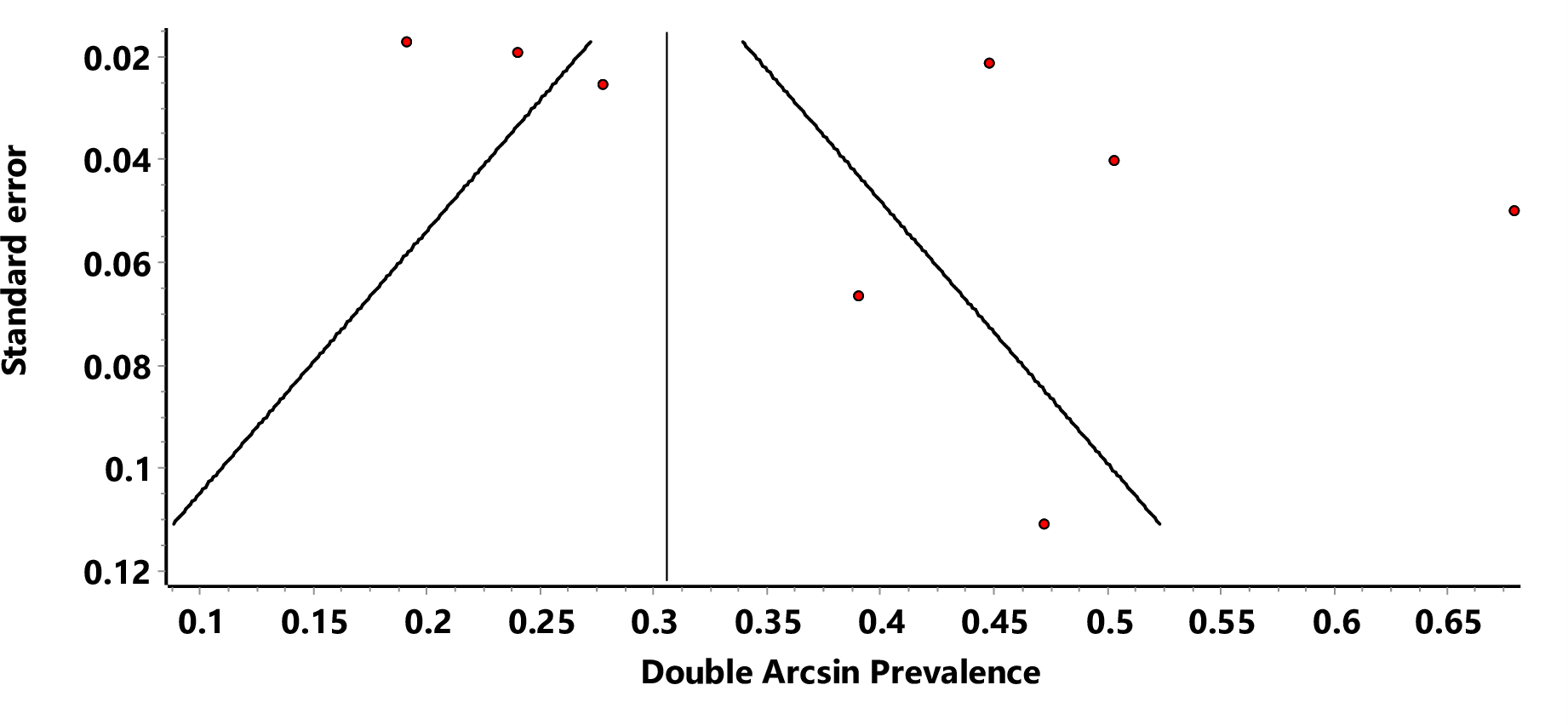

Supplement: Supplementary file 1 [file tropicalmed-07-00034-s001.zip › Supplementary File 4,5,6/Supplementary File 5-3 Funnel plot blindness.tif]

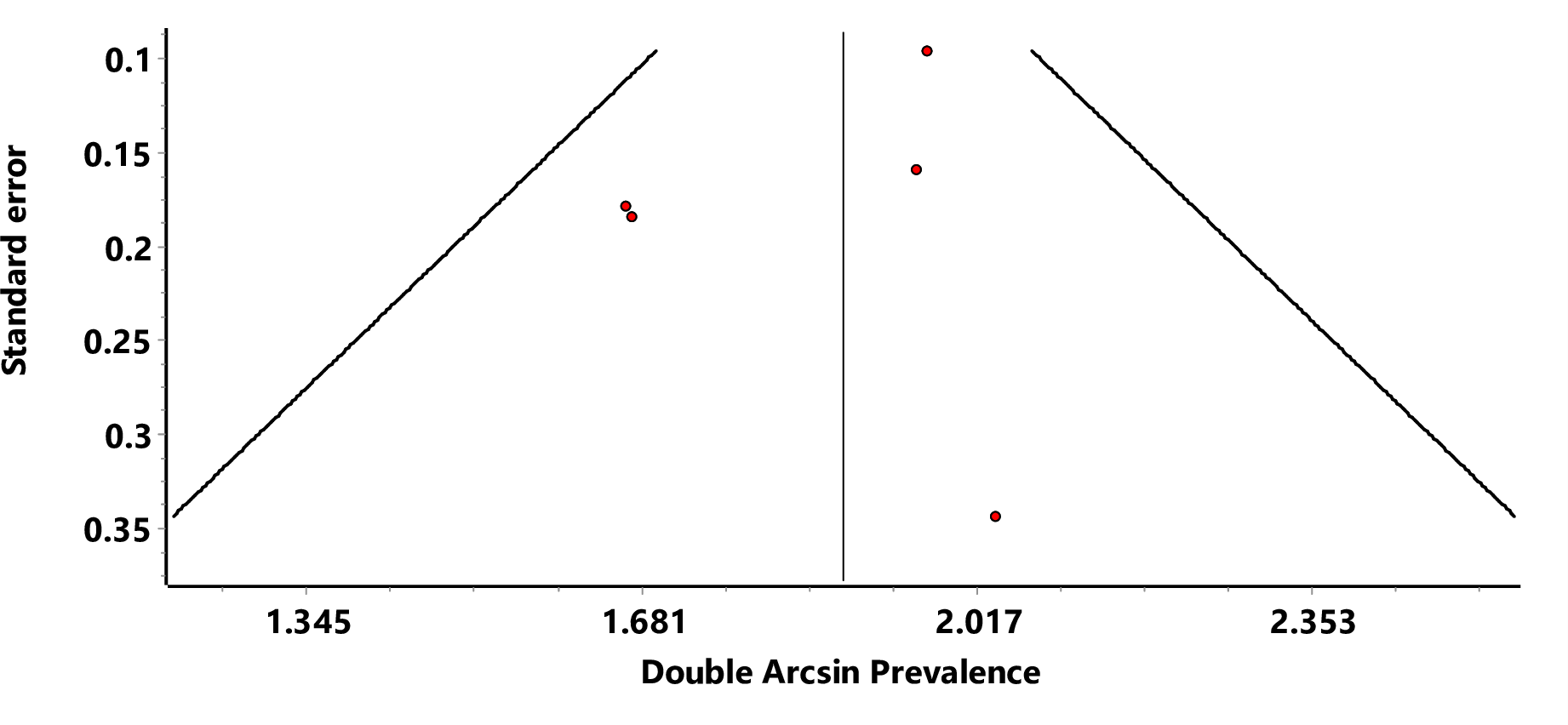

Supplement: Supplementary file 1 [file tropicalmed-07-00034-s001.zip › Supplementary File 4,5,6/Supplementary File 5-4 Funnel plot cataracts Blindness.tif]

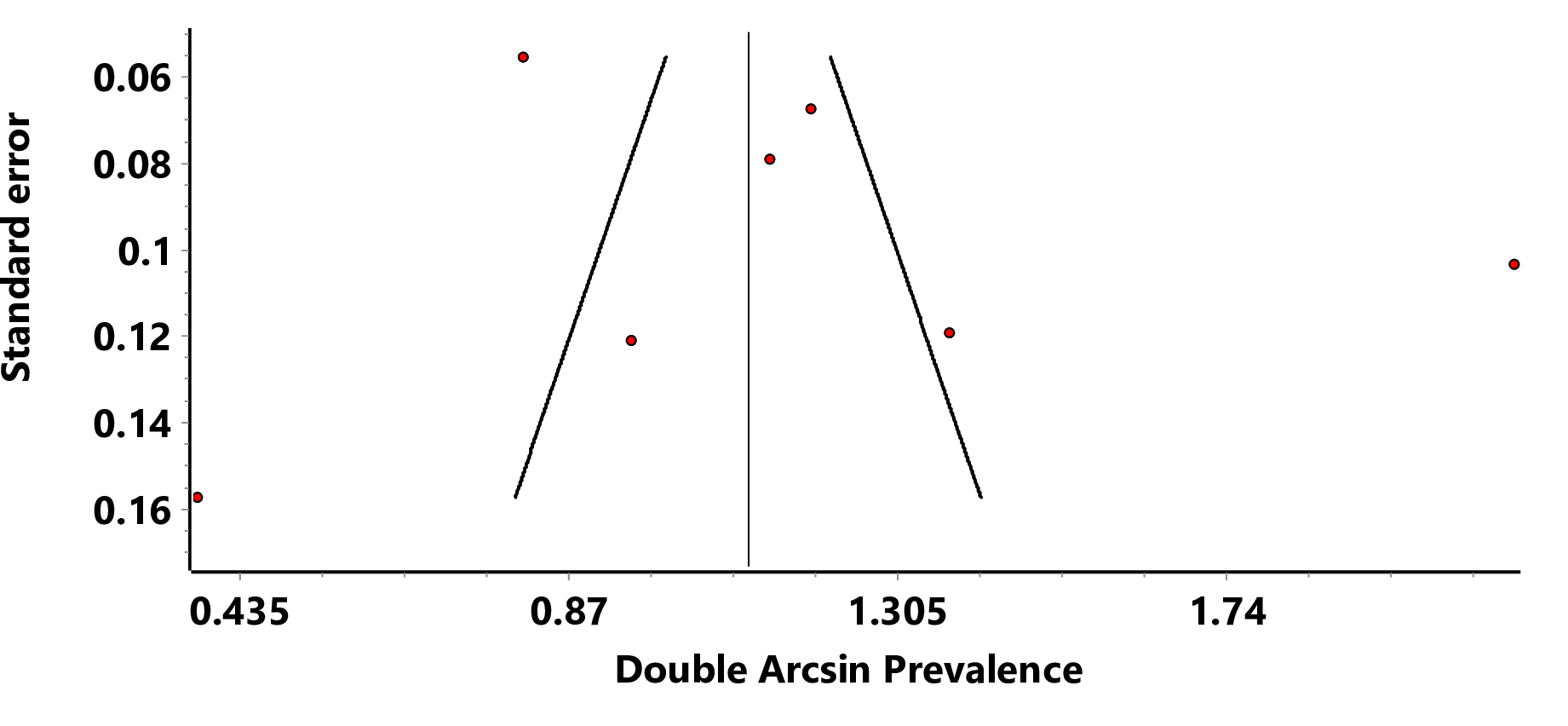

Supplement: Supplementary file 1 [file tropicalmed-07-00034-s001.zip › Supplementary File 4,5,6/Supplementary File 5-5 MSVI cataracts funnel plot.tif]

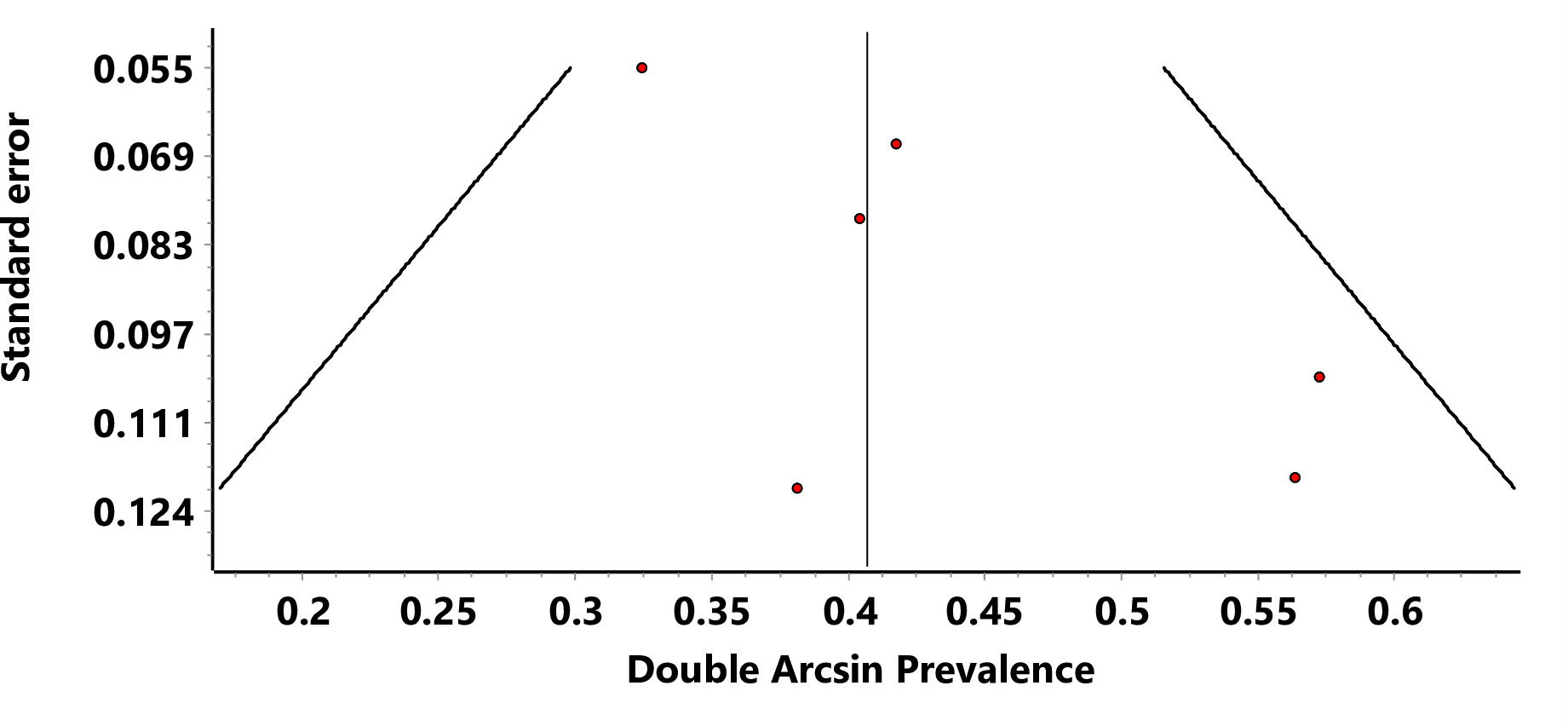

Supplement: Supplementary file 1 [file tropicalmed-07-00034-s001.zip › Supplementary File 4,5,6/Supplementary File 5-6 MSVI Dr funnel plot.tif]

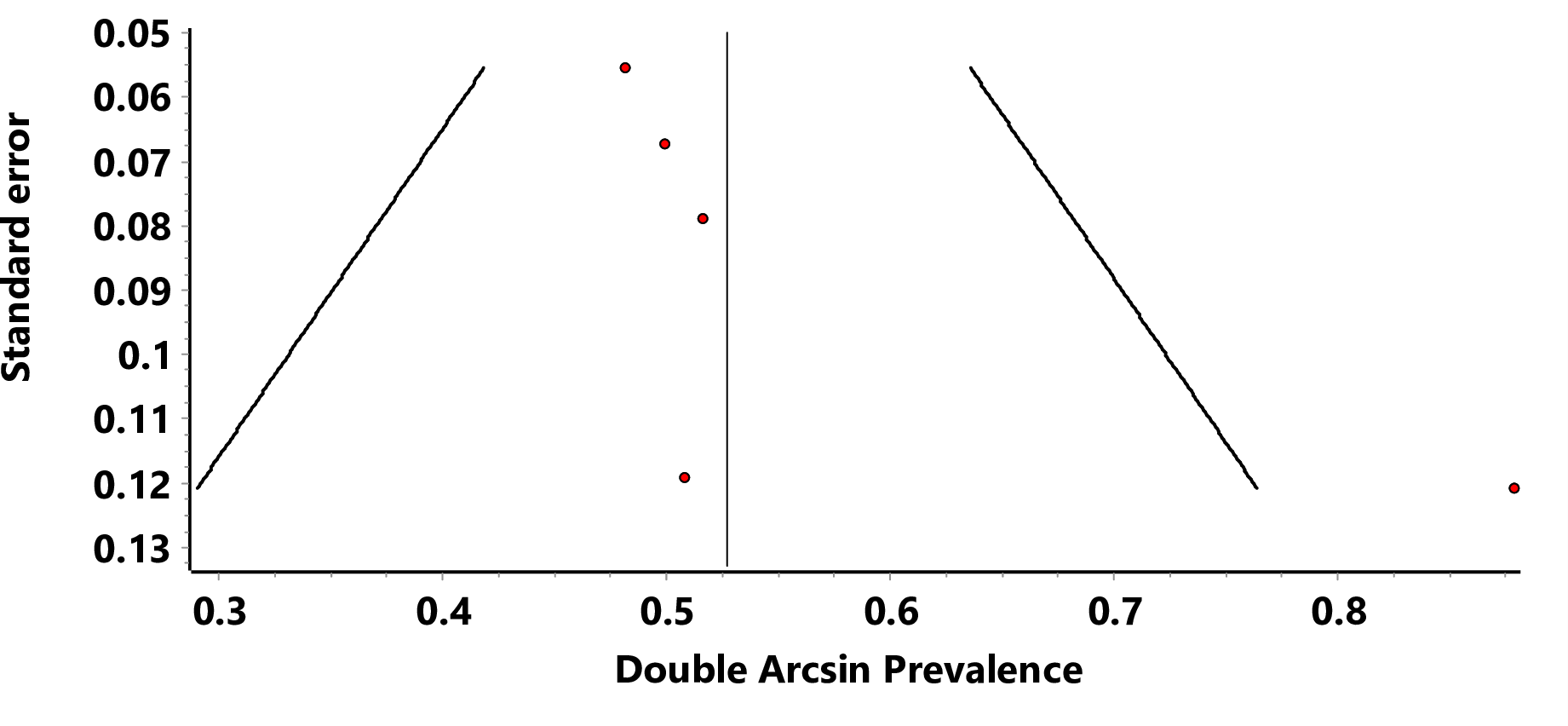

Supplement: Supplementary file 1 [file tropicalmed-07-00034-s001.zip › Supplementary File 4,5,6/Supplementary File 5-7 MSVI Galucoma funnel plot.tif]

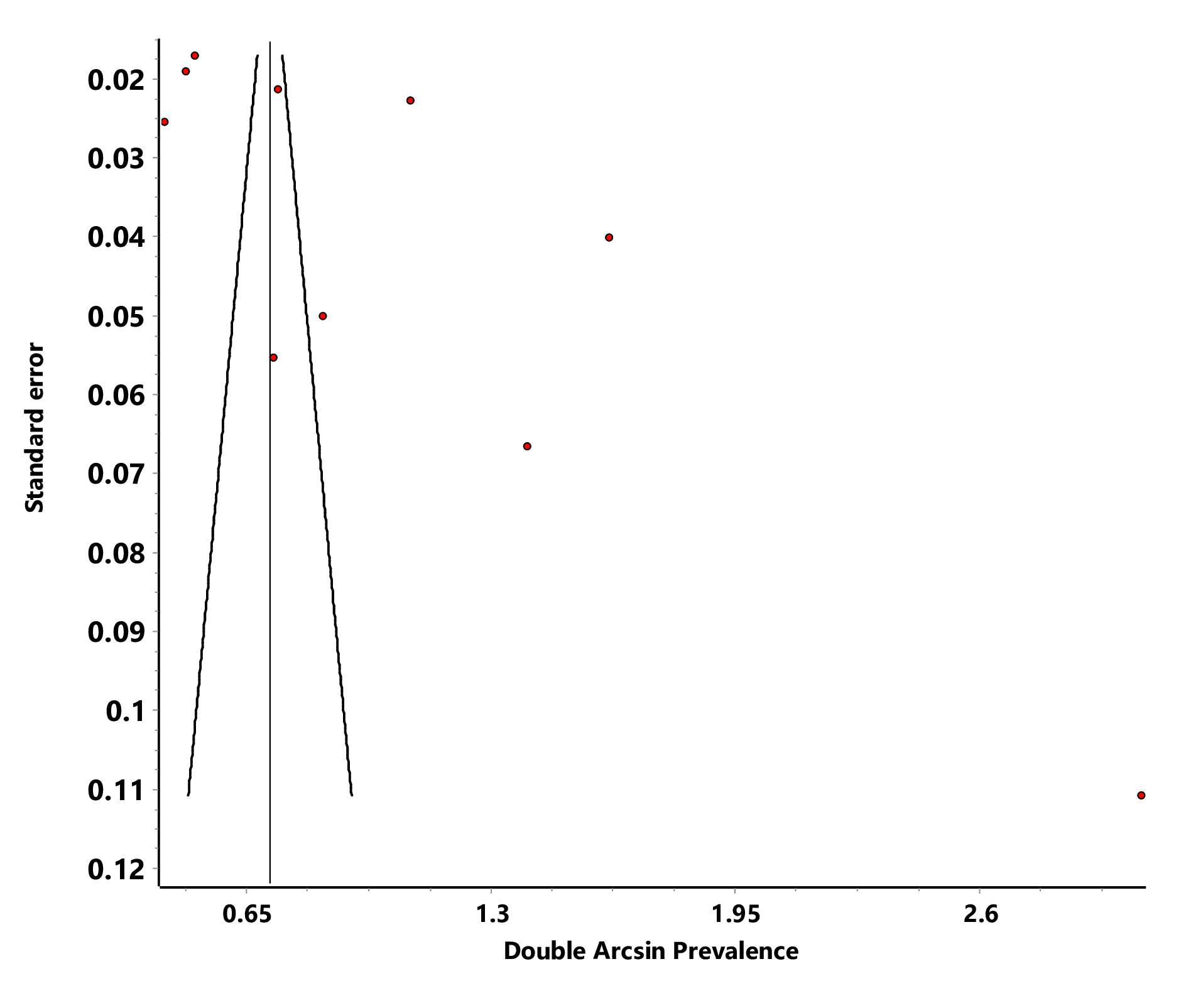

Supplement: Supplementary file 1 [file tropicalmed-07-00034-s001.zip › Supplementary File 4,5,6/Supplementary File 5-8 MSVI prev funnel plot.tif]

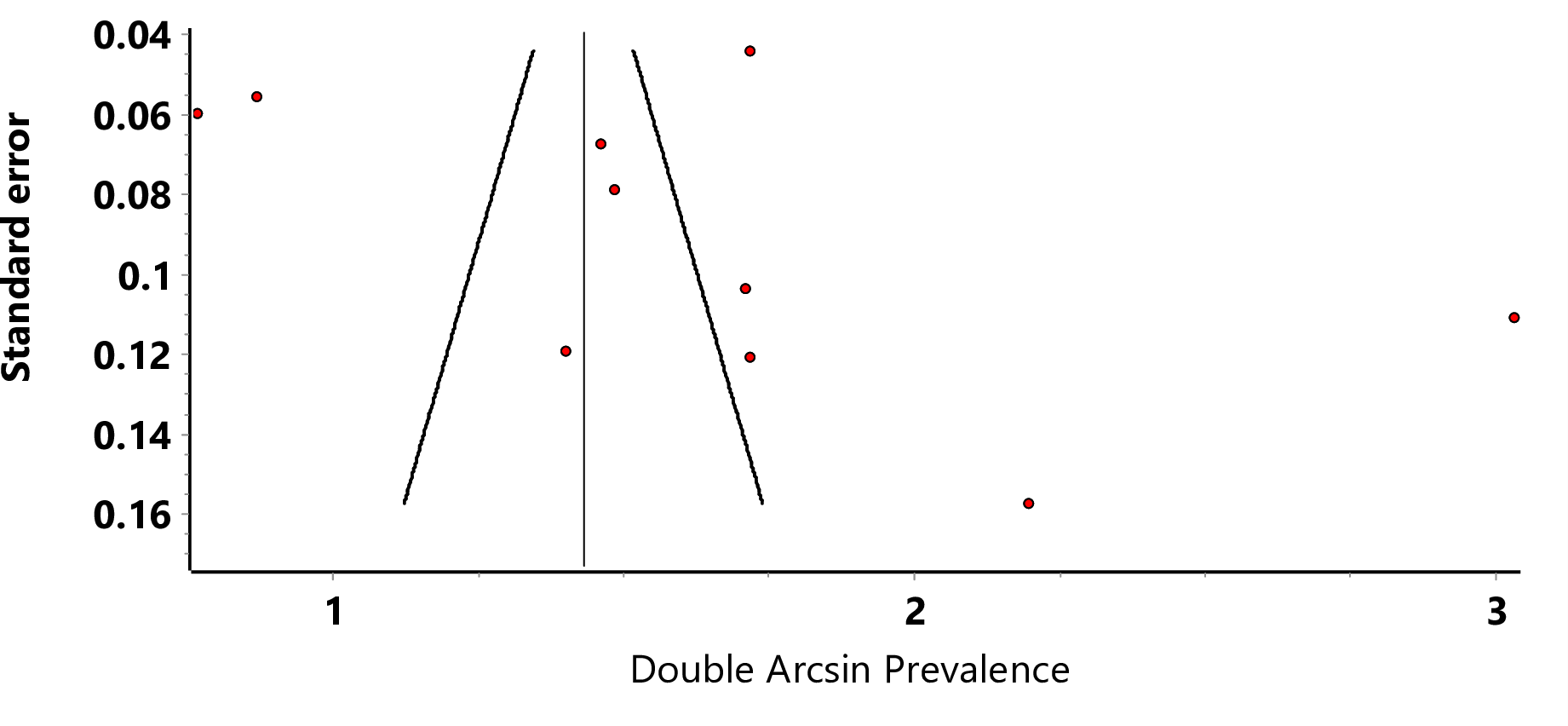

Supplement: Supplementary file 1 [file tropicalmed-07-00034-s001.zip › Supplementary File 4,5,6/Supplementary File 5-9 MSVI URE funnel plot.tif]

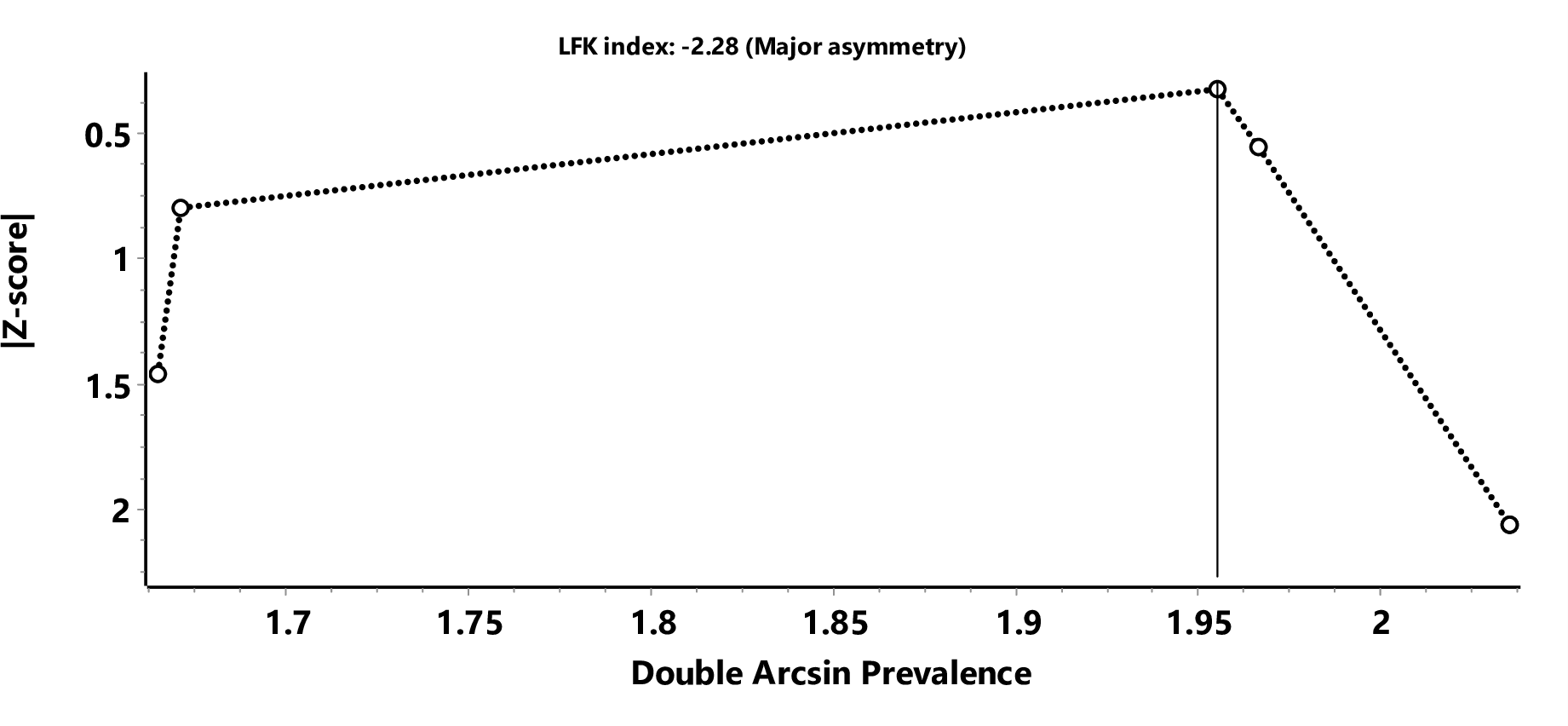

Supplement: Supplementary file 1 [file tropicalmed-07-00034-s001.zip › Supplementary File 4,5,6/Supplementary File 6-1 Doi plot blindness cataracts.tif]

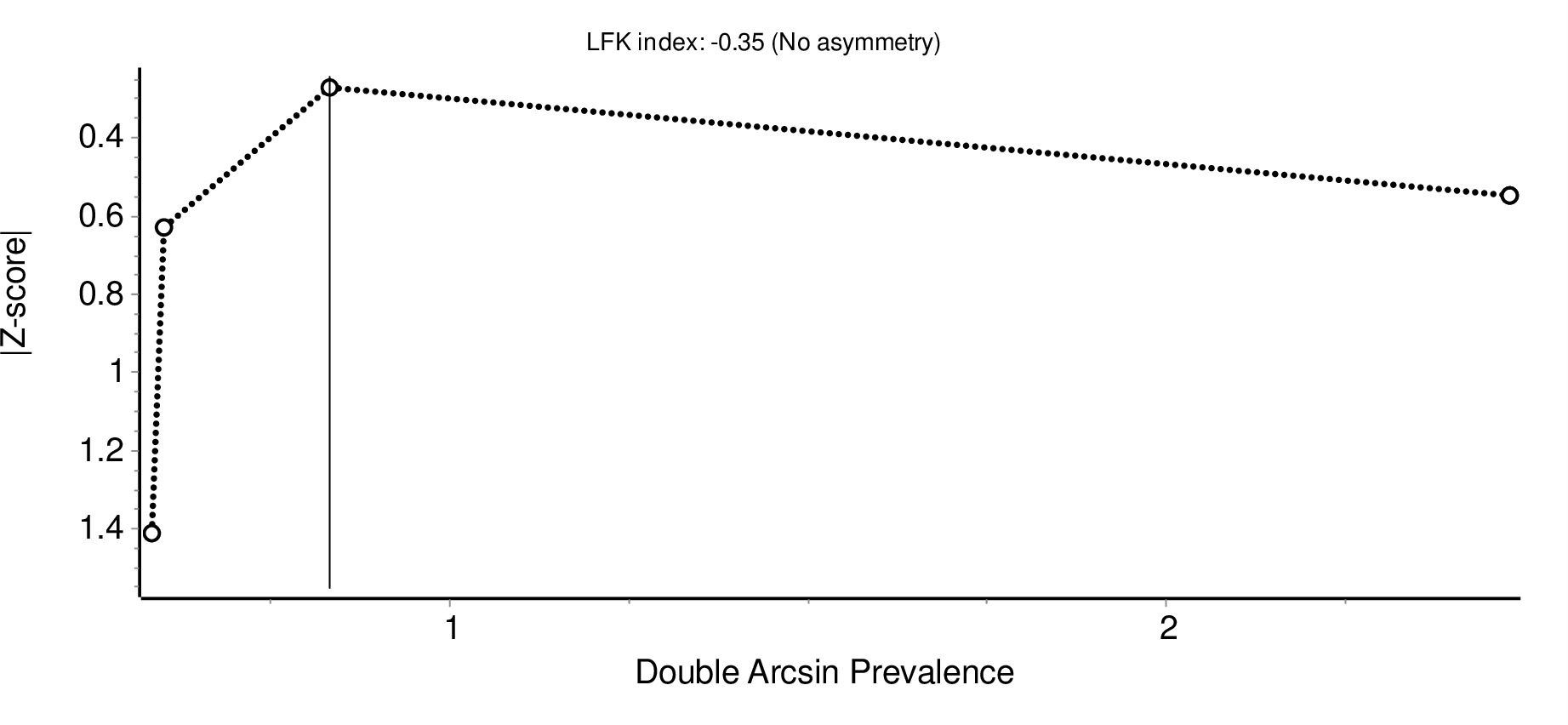

Supplement: Supplementary file 1 [file tropicalmed-07-00034-s001.zip › Supplementary File 4,5,6/Supplementary File 6-2 Doi plot Blindness DR.tif]

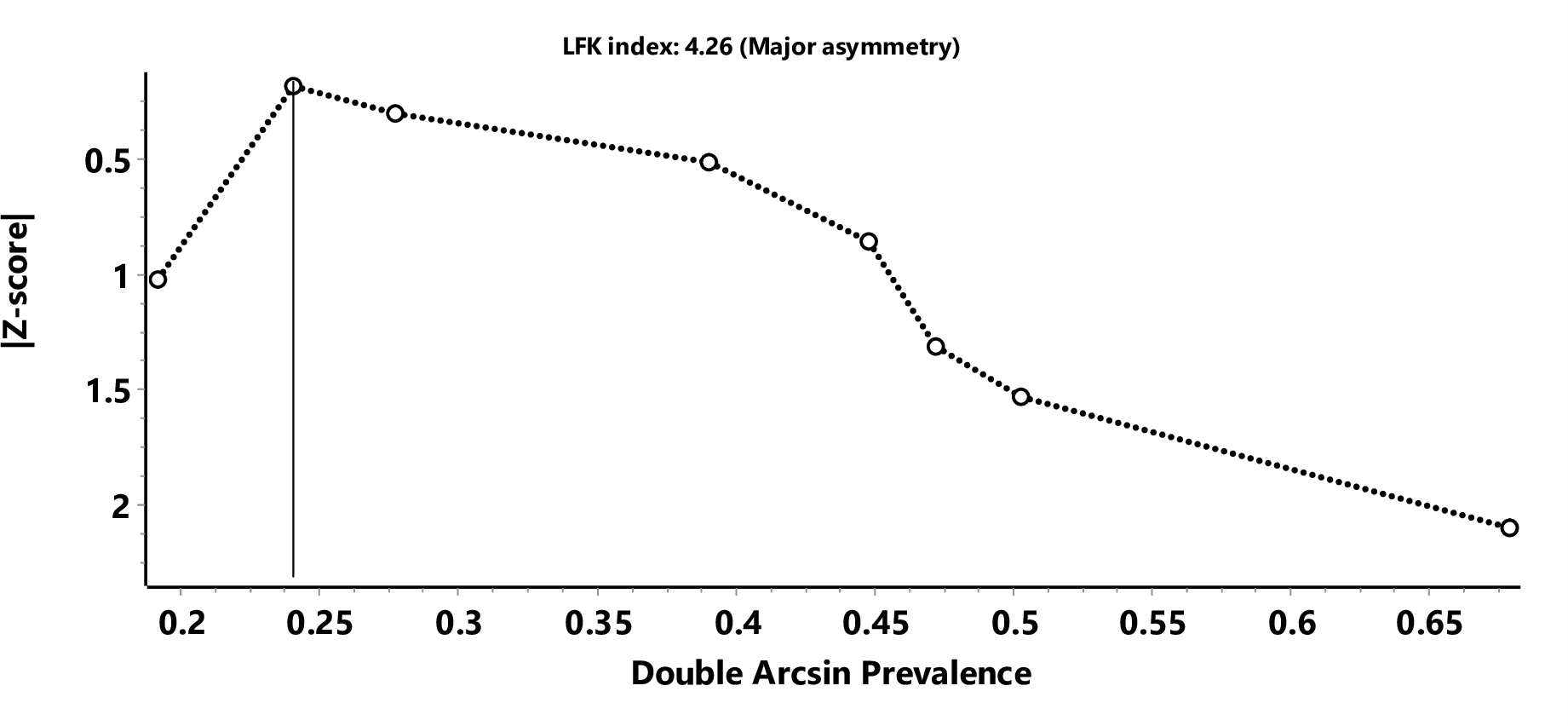

Supplement: Supplementary file 1 [file tropicalmed-07-00034-s001.zip › Supplementary File 4,5,6/Supplementary File 6-3 Doi plot Blindness.tif]

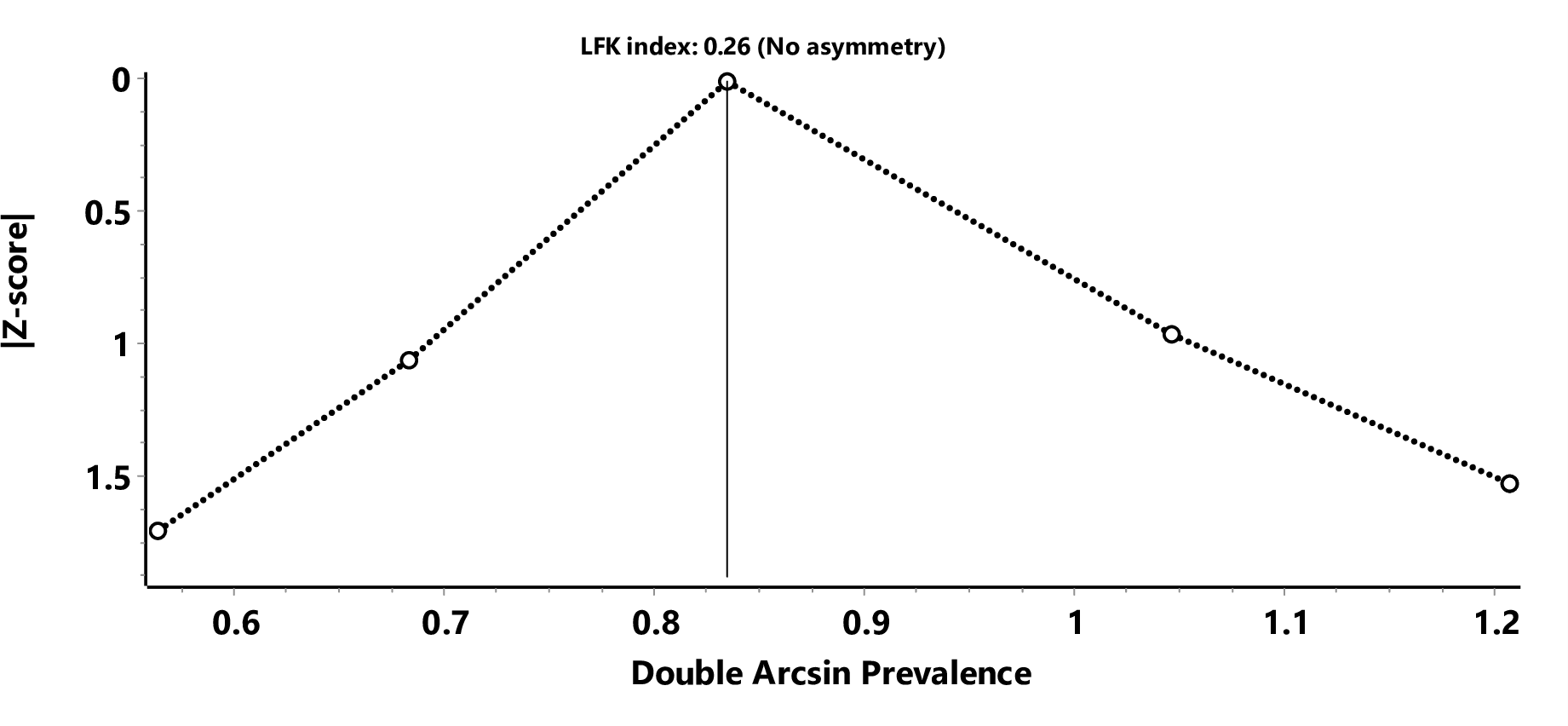

Supplement: Supplementary file 1 [file tropicalmed-07-00034-s001.zip › Supplementary File 4,5,6/Supplementary File 6-4 Doi plots Blindness glaucoma.tif]

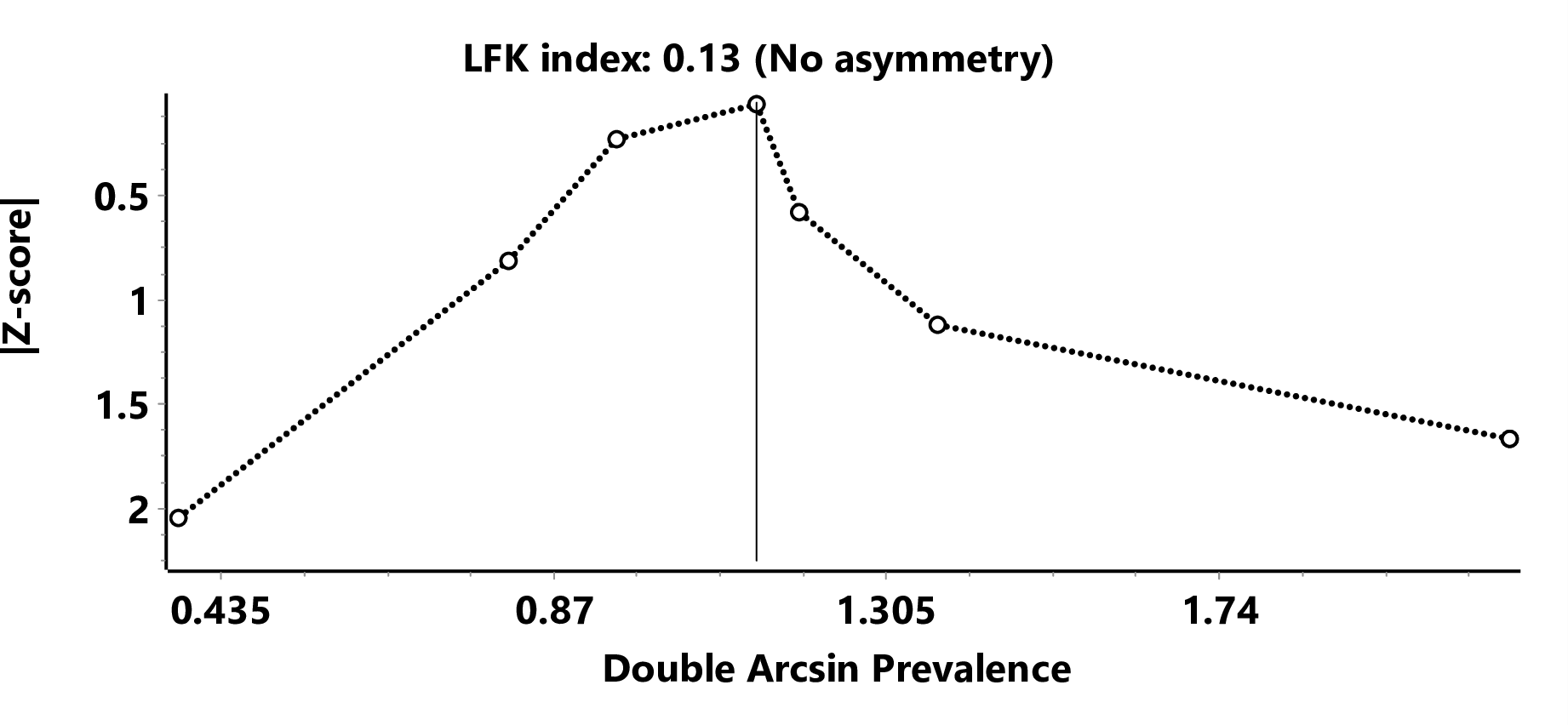

Supplement: Supplementary file 1 [file tropicalmed-07-00034-s001.zip › Supplementary File 4,5,6/Supplementary File 6-5 MSVI cataracts doi plot.tif]

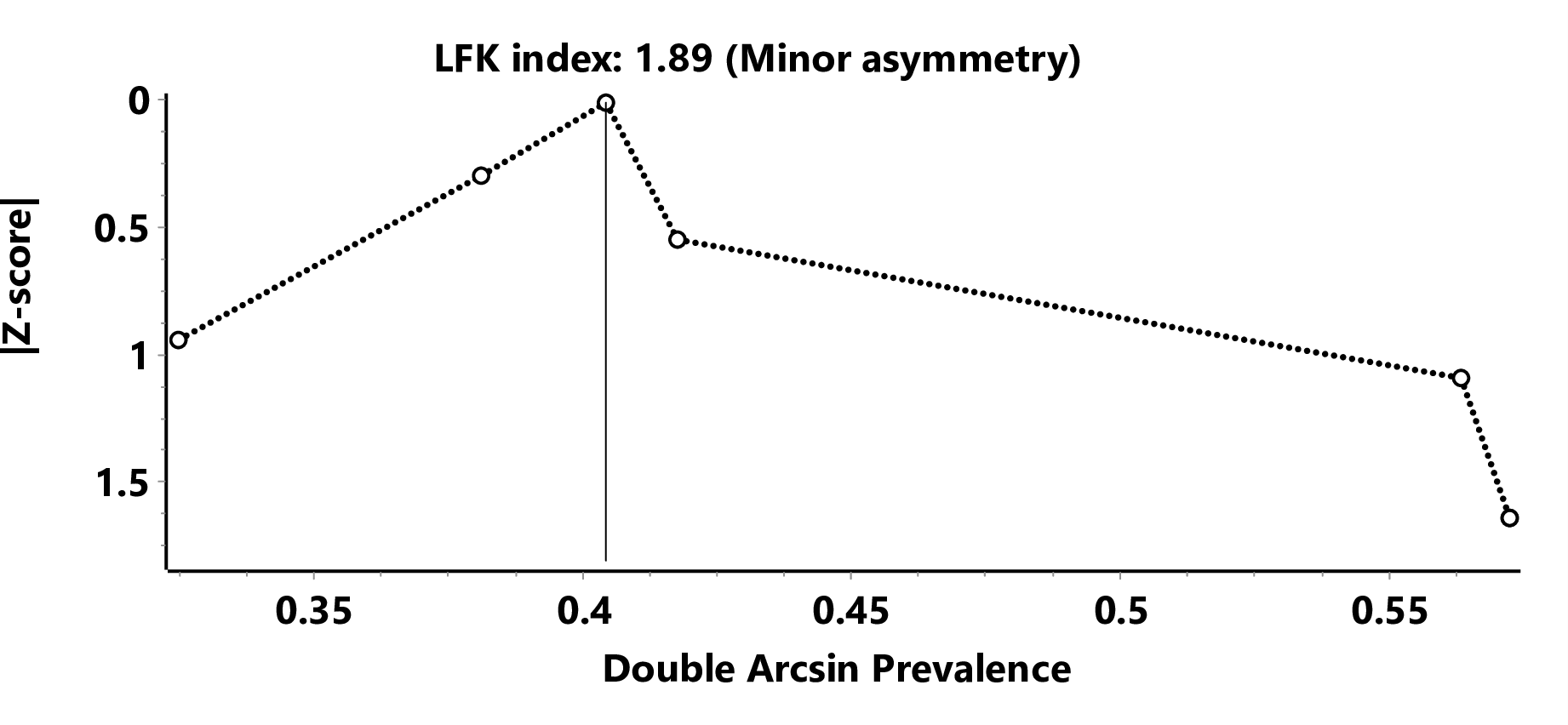

Supplement: Supplementary file 1 [file tropicalmed-07-00034-s001.zip › Supplementary File 4,5,6/Supplementary File 6-6 MSVI DR doi plot.tif]

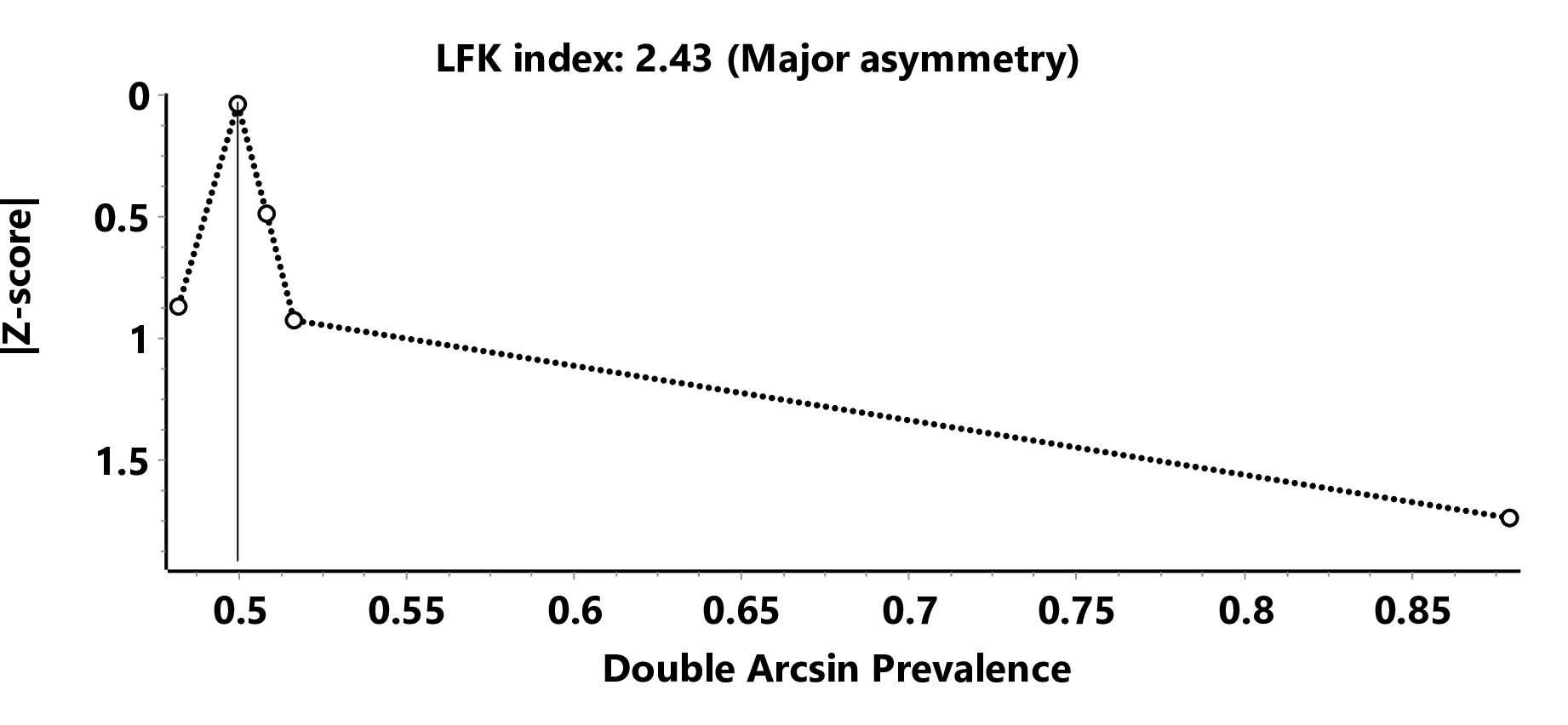

Supplement: Supplementary file 1 [file tropicalmed-07-00034-s001.zip › Supplementary File 4,5,6/Supplementary File 6-7 MSVI Glaucoma doi plot.tif]

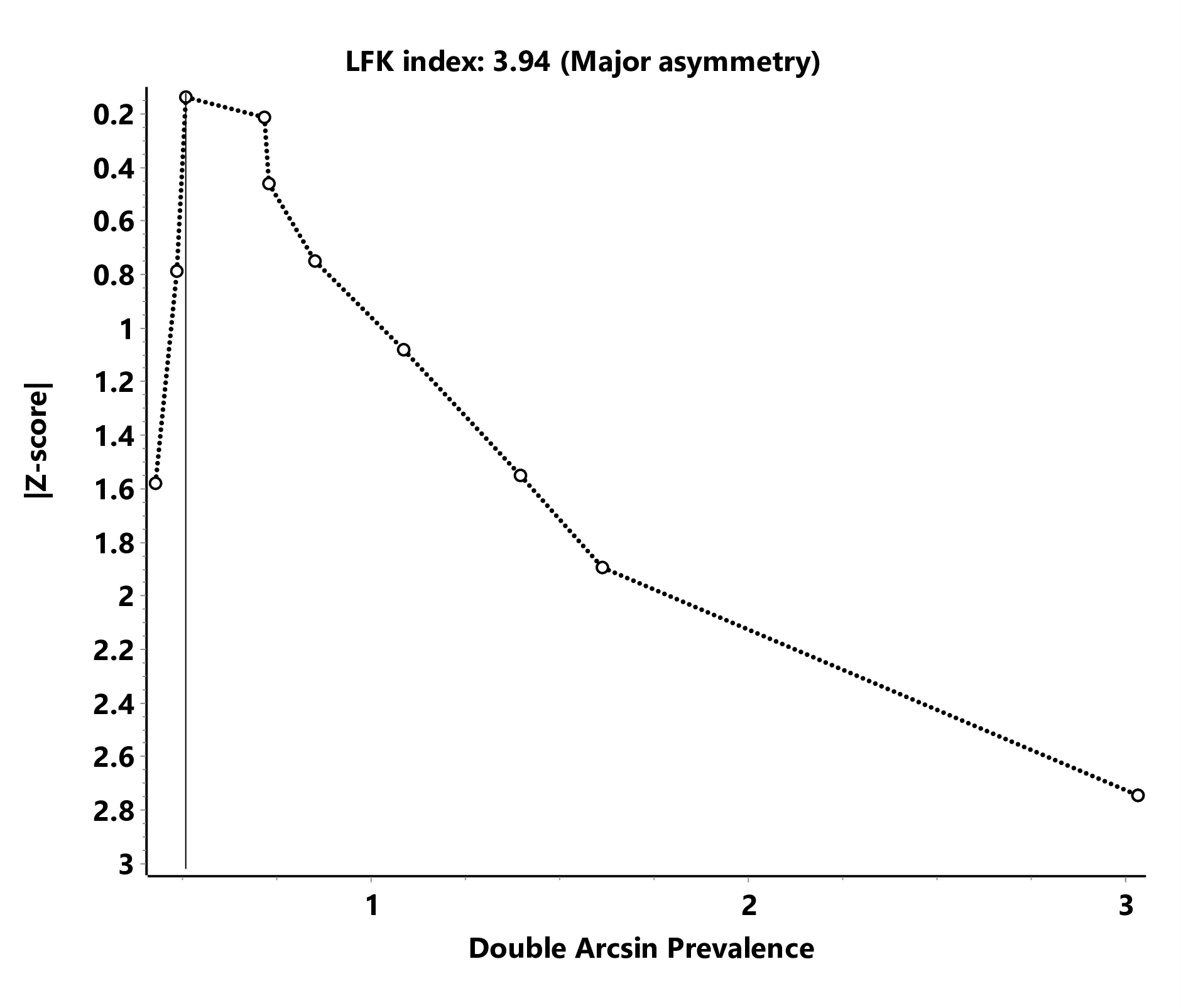

Supplement: Supplementary file 1 [file tropicalmed-07-00034-s001.zip › Supplementary File 4,5,6/Supplementary File 6-8 MSVI Prev doi plot.tif]

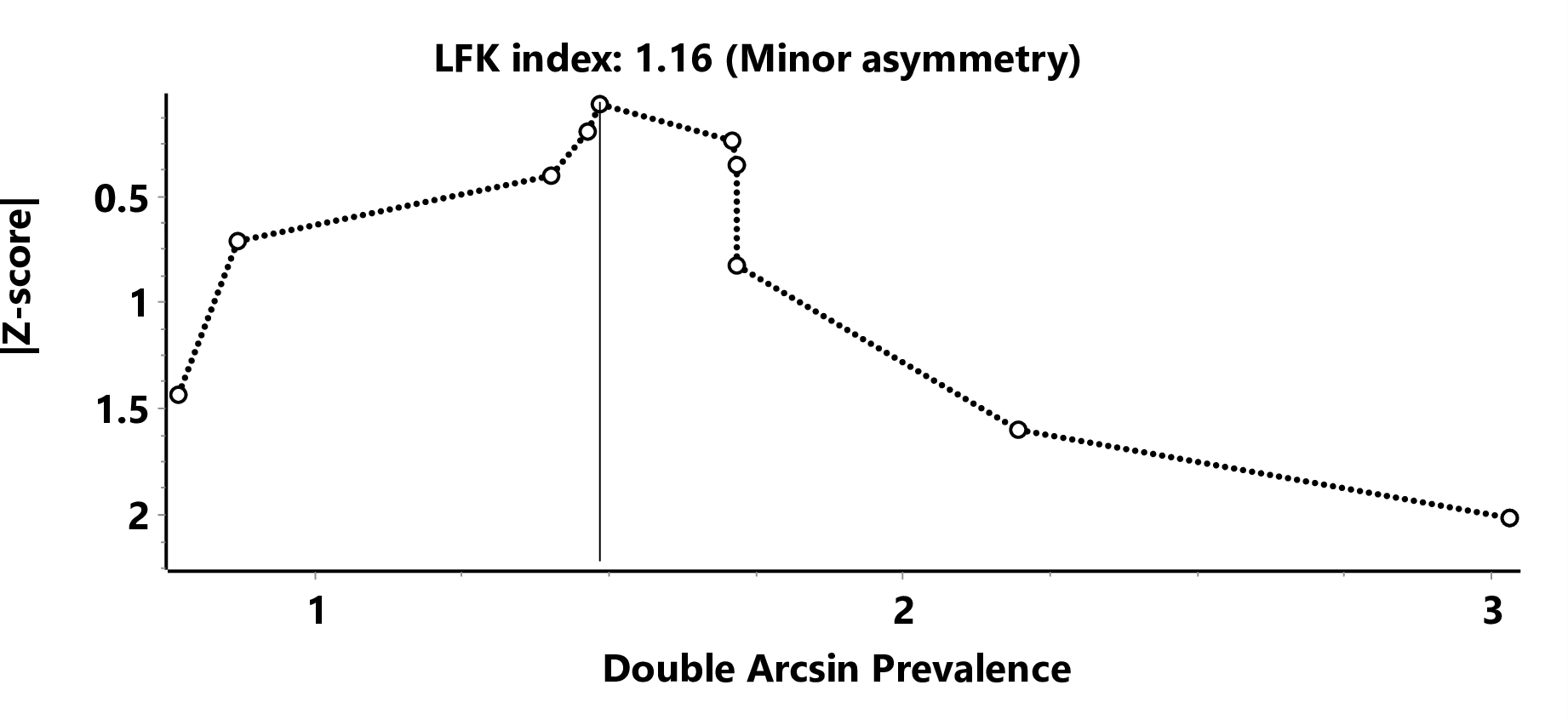

Supplement: Supplementary file 1 [file tropicalmed-07-00034-s001.zip › Supplementary File 4,5,6/Supplementary File 6-9 MSVI URE doi plot.tif]
